# Twist1 contributes to developing and sustaining corticosteroid resistance in ulcerative colitis

**DOI:** 10.7150/thno.62256

**Published:** 2021-06-26

**Authors:** Changqin Liu, Li-Hua Mo, Bai-Sui Feng, Qiao-Ruo Jin, Yan Li, Jianli Lin, Qing Shu, Zhi-Gang Liu, Zhanju Liu, Xiaomin Sun, Ping-Chang Yang

**Affiliations:** 1 Department of Gastroenterology, The Shanghai Tenth People's Hospital of Tongji University, Shanghai, China.; 2 Guangdong Provincial Key Laboratory of Regional Immunity and Diseases, Shenzhen, China.; 3 Research Center of Allergy & Immunology, Shenzhen University School of Medicine, Shenzhen, China.; 4 Department of Gastroenterology, the Second Hospital, Zhengzhou University, Zhengzhou, China.; 5 Department of Gastroenterology, First Affiliated Hospital, Shenzhen University, Shenzhen, China.

## Abstract

**Rationale:** Corticosteroid resistance (CR) is a serious drawback to steroid therapy in patients with ulcerative colitis (UC); the underlying mechanism is incompletely understood. Twist1 protein (TW1) is an apoptosis inhibitor and has immune regulatory functions. This study aims to elucidate the roles of TW1 in inducing and sustaining the CR status in UC.

**Methods:** Surgically removed colon tissues of patients with ulcerative colitis (UC) were collected, from which neutrophils were isolated by flow cytometry. The inflammation-related gene activities in neutrophils were analyzed by RNA sequencing. A CR colitis mouse model was developed with the dextran sulfate sodium approach in a hypoxia environment.

**Results:** Higher TW1 gene expression was detected in neutrophils isolated from the colon tissues of UC patients with CR and the CR mouse colon tissues. TW1 physically interacted with glucocorticoid receptor (GR)α in CR neutrophils that prevented GRα from interacting with steroids; which consequently abrogated the effects of steroids on regulating the cellular activities of neutrophils. STAT3 (Signal Transducer and Activator of Transcription-3) interacted with Ras protein activator like 1 to sustain the high TW1 expression in colon mucosal neutrophils of CR patients and CR mice. Inhibition of TW1 restored the sensitivity to corticosteroid of neutrophils in the colon tissues of a CR murine model.

**Conclusions:** UC patients at CR status showed high TW1 expression in neutrophils. TW1 prevented steroids from regulating neutrophil activities. Inhibition of TW1 restored the sensitivity to corticosteroids in the colon tissues at the CR status.

## Introduction

Ulcerative colitis (UC) is one of the two major phenotypes of inflammatory bowel disease (IBD). The prevalence of UC is about 0.25-0.5% worldwide 1. It is recognized that UC is a chronic inflammatory disease in the colon mucosa (only in severe forms the inflammatory process can also involve the submucosal layer). Profound infiltration of inflammatory cells, *e.g.*, eosinophils and neutrophils, and many inflammatory cytokines in the colon mucosa are pathological features of UC 2. Because the UC pathogenesis is unclear, there are no specific therapies that can cure UC. Currently, most therapies used for UC are non-specific, one of which is steroids, which are usually used to control the active phases of the disease. Administration of steroids is generally effective in controlling the inflammation, but about one-third of UC patients inadequately respond to steroid therapy 3, 4; this phenomenon is designated corticosteroid resistance (CR). Thus, UC patients with CR status must use higher steroid doses and a longer period of steroid administration to control UC symptoms. Therefore, CR UC patients usually get poorer effects of steroid therapy but higher risk of steroid side effects, such as lower resistance to infections (some CR UC patients show concomitant cytomegalovirus infection 5), weight gain, osteoporosis, hypertension, and worsening of diabetes 4, 6. Yet, the mechanism of CR development is not fully understood. Therapies to reverse CR are not currently available 4.

The effects of steroid agents are mediated by the glucocorticoid receptor α (GRα) in the cell. Steroids diffuse into the cytoplasm to activate GRα. GRα then translocates into the nucleus to activate relevant anti-inflammatory genes to render the anti-inflammatory action 6. Another receptor of steroids is the GRβ. GRβ does not bind steroids but occupies relevant target genes to prevent steroids from regulating inflammatory activities 6. It is reported that GRβ expression is higher in CR subjects than in corticosteroid sensitive (CS) subjects 6. However, how the expression of GRβ is upregulated and how CRα is dysfunctional in CR subjects remain elusive.

Neutrophils are the major effector cells contributing to the inflammatory status in the colon mucosa of UC patients 7, 8. Neutrophils are one of the major fractions of white blood cells that play major immune defensive roles in the body. Neutrophils phagocyte invading microbes and senescent cell debris, but over-activated neutrophils release many enzymes or proinflammatory mediators to induce or sustain inflammation in the colon 8. It is the consensus that neutrophils play a central pathogenic role in UC 8; however, effective medical therapies selectively target neutrophils for UC are still lacking 8. Profound neutrophil exudation can be generally observed in the colon mucosa in patients with UC; however, whether neutrophils are associated with the development of the CR status is unknown.

To search possible links between neutrophils and the CR development of UC patients, we analyzed UC colon neutrophils with RNA sequencing (RNAseq). The abundance of Twist1 (TW1) gene expression was high in CR neutrophils. TW1 is one of the apoptosis inhibitors and is expressed by many cancer-cell types, playing a role in cancer pathogenesis, such as contributing to cancer overgrowth and epithelial-mesenchymal transition 9. TW1 also has immune regulatory functions, *e.g.*, regulating macrophage activities, promoting dendritic cell functions, and involving vasculitis in atherosclerosis 10-12. Aberrant activities of macrophages, dendritic cells, and vasculitis also involve UC pathogenesis 2, 13. Whether TW1 is associated with the CR status in UC patients has not been investigated. The RNAseq results also showed high K-Ras (Ras) gene expression in CR neutrophils. Ras is an abbreviation of rat sarcoma, including subtypes of K-Ras, H-Ras, and N-Ras. Ras is involved in the signal transduction pathways of multiple cell activities, one of which is to sustain relevant cell activities, such as cancer cell overgrowth 14. Based on the above information, we hypothesize that TW1 facilitates CR development via regulating neutrophil activities in IBD patients. To this end, we collected surgically removed colon tissues from patients with UC with or without CR. The results showed that CR colon neutrophils expressed high levels of TW1, which prevented neutrophils from responding to the regulatory effects of steroids.

## Materials and methods

### Reagents

Anti-TW1 antibody (Ab; ab49254) and ELISA Kits of Ras GTPase and Ras GDPase were purchased from abcam (Cambridge, MA). TW1 CRISPR/Cas9 KO plasmids, Abs of RASAL1 (B-2), STAT3 (F-2), pSTAT3 (23G5), KRas (F234), CD11b (44; AF488), HRP-His tag (H-3), Ly6G Ab (RB6-8C5; AF594), CD45 (35-Z6, AF790), CD11c (3.9, AF680), CD3 (PS1, AF700), CD19 (B-1, AF594), EpCAM (C-10, AF647) and Farnesyl thiosalicylic acid (FTS) were purchased from Santa Cruz Biotech (Santa Cruz, CA). CD66b Ab (AF647) was purchased from BD Bioscience (Franklin Lakes, NJ). PE-Siglec 8 Ab (7C9) was purchased from BioLegend (San Diego, CA). Reagents and materials for immunoprecipitation, RT-qPCR and Western blotting, GRβ (PA3-514), GRα (PA1-516) Abs were purchased from Invitrogen (Carlsbad, CA). Proteins of IL-6 and TGF-β, ELISA kits for determining ECP, NE, MPO, IL-1β, TNF-α, IFN-γ, IL-6 and IL-17 were purchased from R&D Systems (MinneAFolis, MN). Recombinant (r) KRas, His-rRASAL1, rSTAT3, rTW1, rGR and rCortisol were provided by Sangon Biotech (Shanghai, China). Stattic, EDTA, dexamethasone, dextran sulphate sodium, Cobalt chloride, PMA and collagenase IV were purchased from Sigma Aldrich (St. Louis., MO).

### Human subjects

UC patients with dysplasia in the colon mucosa planned to be treated with surgery were recruited into this study at the IBD Center, Shanghai Tenth People's Hospital of Tongji University from May 2017 to March 2020 (Fig. S1; see below). The UC diagnosis was referred to published procedures 15, 16. A portion of severe UC patients required surgical treatment 17. Recruited patients were divided into CR group and CS (corticosteroid sensitive) group based on a Dex test (dexamethasone test), in which UC patients received systemic administration of dexamethasone (Dex, in short; 4 mg/kg) in a 1-week period referring to previous reports with modifications 18, 19 followed by the gradual Dex dose-reduction. The CS status was defined as UC symptoms improved from severe to moderate mild, or from moderate mild to mild, after Dex test (see below). The CR status was defined if no symptom improvement was resulted from the Dex test. The diagnosis and surgery of UC were carried out by our doctors. Surgically removed colon tissues (the dysplasia area was avoided being sampled) were collected from the operation facilities and processed immediately for further experiments. Surgically removed colon cancer tissues were also collected. The marginal non-cancer colon tissues (proved by pathologists) were used as the non-UC controls (the NC group). Patients treated by anti-cancer therapy were excluded. The study protocol was approved by the Human Ethical Committee at Shanghai Tongji University (#TJHEC2017008). A written informed consent was obtained from each human subject. Patients with toxic megacolon, Crohn's colitis or indeterminate colitis were excluded. The demographic data are presented in Table 1.

**UC symptom scale** (referred to a paper: Truelove SC, Witts LJ. Cortisone in ulcerative colitis; final report on a therapeutic trial. Br Med J. 1955 Oct 29; 2 (4947): 1041-8).

- Severe: (1) severe diarrhea (6 or more times/day); (2) apparent blood in stools; (3) fever (mean evening temperature more than 37.5 °C); (4) tachycardia (pulse rate > 90/min); (5) anemia; (6) E.S.R. much raised (more than 30 mm/h).
- Mild: (1) mild diarrhea (four or less times a day); (2) no blood or only small amounts of blood in stools; (3) no fever; (4) no tachycardia; (5) anemia not severe; (6) E.S.R. not raised above 30 mm/h.
- Moderately severe: Intermediate between severe and mild.

### Histology

A piece of colon tissue was fixed with 4% formalin overnight and processed for paraffin sections. After staining with hematoxylin and eosin, the sections were observed with a light microscope.

### Fluorescence activating cell sorting (FACS)

Single cells were prepared with the colon tissues. In the surface staining, cells were stained with antibodies (labeled with fluorochromes) of interest (diluted at 1:100) or isotype IgG for 30 min on ice. After washing with phosphate-buffered saline (PBS) 3 times, the cells were analyzed with a flow cytometer (FACSCanto II, BD Bioscience). In the intracellular staining, cells were fixed with 1% paraformaldehyde (containing 0.05% Triton X-100) for 1 h, washed with PBS 3 times, and then subjected to the surface staining procedures. The results were analyzed with Flowjo (Tree Star Inc., Ashland, OR). Data from isotype IgG staining were used as the gating references (Fig. S2).

### Isolation of immune cells from the colon tissues

Surgically removed colon tissues were collected and cut into small pieces (about 2 × 2 × 2 mm). Samples were incubated in a solution containing collagenase IV (1 mg/ml) and EDTA (Ethylenediaminetetraacetic acid; 0.05%) for 30 min with mild agitation at 37 °C. Single cells were collected by filtering through a cell strainer (100 µm first, then 70 µm) and followed by centrifugation at 1000 ×*g* for 5 min. As shown by Fig. S2, CD45^+^ cells were gated first, then immune cells of interest [Neutrophils (CD11b^+^ CD66b^+^), eosinophils (CD11b^+^ siglec 8^+^), DCs (CD11c^+^), T cells (CD3^+^) and B cells (CD19^+^)] were purified from CD45^+^ cells by Fluorescence activating cell sorting (FACS). Epithelial cells were purified from the single cells with EpCAM as the cell marker. Purity of isolated cells were greater than 95% as checked by FACS. Viability of isolated cells was greater than 99% as assessed by the Trypan blue exclusion assay.

### RNA sequencing (RNAseq)

Purified neutrophils were processed for RNA extraction using the TRIzol reagents. RNA samples were sent to EGene Biotech (Shenzhen, China). All the procedures of RNAseq and data processing were carried out by the professional staff of the company. Briefly, by using an RNA library kit (TruSeq RNA library prep kit v2; Illumina), the RNA sequencing libraries were prepared. Libraries were sequenced using an Illumina HiSeq 2500 sequencer. RNA-seq read quality was assessed using FastQC. The differentially expressed gene (DEG) levels were presented as a heatmap. The raw data are attached in online supplemental materials.

### Real-time quantitative RT-PCR (RT-qPCR)

Neutrophils were prepared as described above. RNA was extracted from cells with TRIzol reagents following manufacturer's instruction, and then converted to cDNA with a reverse transcription kit according to the manufacturer's instruction. The cDNA was amplified in a qPCR device (CFX96 Touch Real-Time PCR Detection System) with SYBR Green Master Mix and in the presence of TW1 primers (ttccgagttctatgccctgg and ccagtttacgatgcagagcc) or KRas (agtgccttgacgatacagct and cctccccagtcctcatgtac). The results were calculated with the 2^-∆∆Ct^ method and presented as relative quantification.

### Western blotting

Proteins (50 µg/well) were fractioned by SDS-PAGE (sodium dodecyl sulphate-polyacrylamide gel electrophoresis), transferred onto a polyvinylidene fluoride (PVDF) membrane. After incubating with 5% skim milk solution for 30 min to block non-specific binding, the membrane was incubated with primary antibodies of interest (diluted in 1:500) overnight at 4 °C with gentle rotation, washed with TBST (Tris-buffered saline containing 0.05% Tween 20) 3 times, incubated with horseradish-labeled secondary antibodies (diluted in 1:5000) for 2 h at room temperature, washed with TBST 3 times. The immunoblots on membranes were developed with the enhanced chemiluminescence and photographed in the GelDoc Go image station (Bio-Rad).

### Enzyme-linked immunosorbent assay (ELISA)

Cytokine levels in samples were determined by ELISA with commercial reagent kits following the manufacturer's instructions.

### Depletion of TW1 in neutrophils

The TW1 expression in purified neutrophils was depleted by the CRISPR (clustered regularly interspaced short palindromic repeats) approach with commercial reagent kits following the manufacturer's instruction, in which neutrophils were electroporated using a Bio-Rad Gene Pulse set at 950 μF and 280 V.

### Treating mice with hypoxia

Following published procedures 20 with modification, KO mice (mice with TW1-deficient neutrophils) and wild-type (WT) mice were exposed to normobaric hypoxia (10% O_2_) or normoxia for 1 week. Briefly, mice were placed in a chamber and treated with a mixture of 10% O_2_ and 90% N_2_. Normoxic mice were maintained in room air (21% O_2_). After 1 week of treatment with hypoxia (10% O_2_) or normoxia, mice were used in further experiments.

### Treating cells with hypoxia *in vitro*

Following published procedures 21, cells were cultured in complete RPMI1640 medium at 10^6^ cells/ml. Cobalt chloride (CoCl_2_) was added to the culture at 100 µM. Cells were harvested 24 h later and analyzed in further experiments.

### Mice

BALB/c mice (6-8-week-old) were purchased from the Guangdong Experimental Animal Center (Guangzhou, China). Mice were maintained in a specific pathogen-free facility and allowed to access food/water freely. The experimental protocol was approved by the Animal Ethical Committee at Shenzhen University (SZUAEC2017011).

### Cell culture

Cells were cultured in RPMI1640 medium. The medium was supplemented with 10% fetal serum albumin, 2 mM glutamine, 0.1 mg/ml streptomycin and 100 U/ml penicillin. The supplemental reagents were purchased from Sigma Aldrich.

### Mass spectrometry (MS)

Proteins precipitated by IP were further analyzed by MS. The precipitated proteins were sent to the MS Center at Shenzhen University. The sample analysis and data process were carried out by the professional staff at the center.

### Protein extraction from cells

Cells were lysed with a lysis buffer (1.5 mM MgCl_2_; 0.5 mM DTT; 10 mM KCl; 10 mM HEPES; 0.05% NP40; 1 mM EDTA and protease inhibitor cocktail). Lysates were centrifuged at 13,000 *g* for 10 min. Supernatant was collected and saved as the cytosolic proteins. The pellets were resuspended in a nuclear lysis buffer (0.2 mM EDTA; 1.5 mM MgCl_2_SO_4_; 4.6 M NaCl; 0.5 mM DTT; 5 mM HEPES; 26% glycerol) and incubated for 30 min. Lysates were centrifuged at 13,000 *g* for 10 min. Supernatant was collected and saved as the nuclear proteins. All the procedures were performed at 4 °C.

### Co-immunoprecipitation (co-IP)

Proteins were extracted from neutrophils, incubated with protein G agarose beads for 2 h to adsorb pre-existing immune complexes, followed by centrifugation at 5,000 *g* for 5 min to remove the beads. Supernatant was collected and incubated with Abs (1 µg/ml) of interest or isotype IgG overnight; the samples were then incubated with protein G agarose beads for 2 h to precipitate the immune complexes; the beads were collected by centrifugation at 5,000 *g* for 5 min. Proteins on beads were eluted with an eluting buffer (pH 8.5, 10 mM Tris-Cl) and analyzed by Western blotting.

### Assessment of Ras activation in colon neutrophils

Neutrophils were isolated from the colon tissues and lysed with a lysis buffer (1.5 mM MgCl_2_; 10 mM KCl; 0.5 mM DTT; 10 mM HEPES; 1 mM EDTA; 0.05% NP40 and protease inhibitor cocktail). Samples were incubated with protein G agarose beads for 2 h at 4 °C and centrifuged at 13,000 *g* for 5 min to remove the pre-existing immune complexes. Supernatant was then incubated with an anti-Ras Ab (1 µg/ml) for 2 h at 4 °C. The formed complexes of Ras/anti-Ras Ab were precipitated by incubating with protein G sepharose beads for 2 h at 4 °C; followed by centrifugation at 13,000 *g* for 5 min. The beads were added with an eluting buffer (10 mM Tris-Cl, 1 mM EDTA, pH 8.0) to elute proteins on the beads. GTP and GDP in the precipitated Ras proteins were separated by adding to a TrisPO_4_/EDTA/DTT solution to be heated to 100°C for 3 min. The levels of GTP and GDP in the samples were determined by the GTP ELISA and GDP ELISA, respectively, with specific reagent kits according to the manufacturer's instructions. The Ras activation status was determined by the formula, Ras-bound GTP/(Ras-bound GTP + Ras-bound GDP) × 100 as reported by Scheele et al. 22.

### Assessment of STAT3 binding RASAL1 with competitive ELISA

The recombinant (r) Ras, rSTAT3 and rRASAL1 proteins were provided by the Sangon Biotech (Shanghai, China). A microplate was coated with rRas (100 µl/well; 10 µg/ml) in a coating buffer (anhydrous Na_2_CO_3_, 1.5 g; anhydrous NaHCO_3_, 2.93 g; distilled water, 1 liter; pH 9.6). The plate was incubated at 4 °C overnight, washed with wash buffer (PBS containing 0.05% Tween 20) 3 times, BSA solution (PBS containing 1% w/v BSA) 150 µl was added to each well and incubated at 37 °C for 1 h to block non-specific binding. A solution containing rRASAL1-His (1 µg/ml) and rSTAT3 (diluted to 1:50, 1:100, 1:200, 1:400, 1:800, 1:1600 and 1:3200, respectively) was added to plate (100 µl/well), incubated at 37 °C for 1 h and washed with wash buffer 3 times. An anti-His Ab (HRP-conjugated; diluted in 1:5000) was added to each well (100 µl/well), and incubated at 37 °C for 1 h, washed with wash buffer 3 times. TMB (Tetramethylbenzidine) was added to the plate at 100 µl/well, incubated for 30 min at room temperature. H_2_SO_4_ (0.2 M; 50 µl/well) was added to terminate the reaction. The absorbance at 450 nm was measured with a microplate reader (BioTek™ Synergy™ HTX Multi-Mode Microplate Reader; Thermo Fisher Scientific).

### Assessment of TW1 and cortisol competitively bind glucocorticoid receptor by competitive ELISA

The rTW1, rCortisol-His and rGR (glucocorticoid receptor) proteins were provided by the Sangon Biotech (Shanghai, China). A microplate was coated with rGR (100 µl/well; 10 µg/ml) in a coating buffer. The plate was incubated at 4 °C overnight, washed with wash buffer (PBS containing 0.05% Tween 20) 3 times, BSA solution 150 µl was added to each well and incubated at 37 °C for 1 h to block non-specific binding. A solution containing rCortisol-His (1 µg/ml) and rTW1 (from 1 µg/ml, diluted to 1:50, 1:100, 1:200, 1:400, 1:800, 1:1600 and 1:3200, respectively) was added to plate (100 µl/well), incubated at 37 °C for 1 h and washed with wash buffer 3 times. An anti-His Ab (HRP-conjugated; diluted in 1:5000) was added to each well (100 µl/well), and incubated at 37 °C for 1 h, washed with wash buffer 3 times. TMB (Tetramethylbenzidine) was added to the plate at 100 µl/well, incubated for 30 min at room temperature. H_2_SO_4_ (0.2 M; 50 µl/well) was added to terminate the reaction. The absorbance at 450 nm was measured with a microplate reader.

### Generation of mice carrying TW1-deficient neutrophils

By the genetic engineering approach, the gene of TW1 in neutrophils were conditionally knocked out in BALB/c mice. The TW1 gene was specifically deleted from neutrophils with ELANE as the specific marker to avoid affecting the role of TW1 in other cells. Referring to published procedures 23-25, mice with loxP-flanked TW1 gene (flox) were developed. A gene targeting vector containing three loxP sites was constructed, in which two of them flanking the neomycin resistance gene. The genomic locus was modified between vector and the TW1 gene in embryo stem (ES) cells by homologous recombination. The loxP-flanked TW1 gene was targeted by ELANE-Cre expression in ES cells. Using the modified ES cells, a loxP-TW1 containing mouse line was generated. Then, the mouse strain harboring two loxP sites in the TW1 gene was crossed with another strain expressing ELANE-Cre recombinase. Thus, only in cells expressing ELANE-Cre the TW1 gene becomes inactivated but remained active in other cells of the body. To keep ELANE active until the experimental period, the Cre gene was modified to be functional only in the ribosomes, but could not penetrate the nuclear membrane. Thus, the loxp-TW1 gene was remained intact in the mice. A control mouse strain was also constructed; the mice carried a WT TW1 allele but expression inducible Cre (the cKO mice). Five days prior to experiments, the mice were treated with tamoxifen citrate salt (Sigma Aldrich, St. Louis, MO; 0.3 g/kg in 0.2 ml 10% Etoh in corn oil) by gavage-feeding daily. Control mice received oral gavage of 0.2 ml 10% EtoH in oil. Tamoxifen increases the permeability of the nuclear membrane to allow the ELANE-Cre penetrate the nuclei to cleave the flox-TW1 gene. As assessed by RT-qPCR and Western blotting, all ELANE^+^ neutrophils in the colon tissues did not express TW1 (Fig. S9). Overall, the mice did not show any unusual signs.

### Development of a murine UC model with CR

Following established procedures 26, BALB/c mice were treated by ingestion of 3.5% dextran sulphate sodium (DSS) in drinking water in a hypoxia environment (see above) for 7 days. Colitis was assessed with established procedures 26, including recording the body weight of each mouse every other day; examining colon histology, assessing inflammatory cytokine levels in colon protein extracts by ELISA. To assess the CR status in mice, from day 8 to day 14, colitis mice were treated with dexamethasone (Dex; 5 mg/kg, i.p.; saline was used as a control) daily. Mice were sacrificed on day 15. Colitis parameters were assessed for each mouse. The expected results were that treating colitis mice with Dex would significantly improve the body weight loss and colon histology as well as reducing inflammatory cytokine levels in the colon tissues in mice treated DSS, but not in mice treated with DSS/hypoxia, the latter was expected to develop the CR status. To assess if the established CR could be reversed, from day 8 to day 14, DSS/hypoxia-treated mice were treated with Dex/harmine (a TW1 inhibitor; 10 mg/kg, i.p.) daily. Mice were sacrificed on day 15. Colitis parameters were assessed.

### Statistics

The data are presented as mean ± SEM or median (IQR). In animal experiments, each group consisted of 6 mice. Each experiment was repeated at least 3 times with each sample tested in triplicate (the average of 3 readouts from one sample was used as one datum). The difference between two groups was determined by the Student *t*-test or Mann Whitney test. ANOVA followed by the Dunnett's test or Bonferroni test was performed for multiple comparisons. Correlation between two groups was determined by the Pearson correlation coefficient test or Spearman's rank correlation test. P<0.05 was set as a significant criterion.

## Results

### The colon tissues in UC patients with CR show neutrophil-dominant inflammation

**Figure 1:**
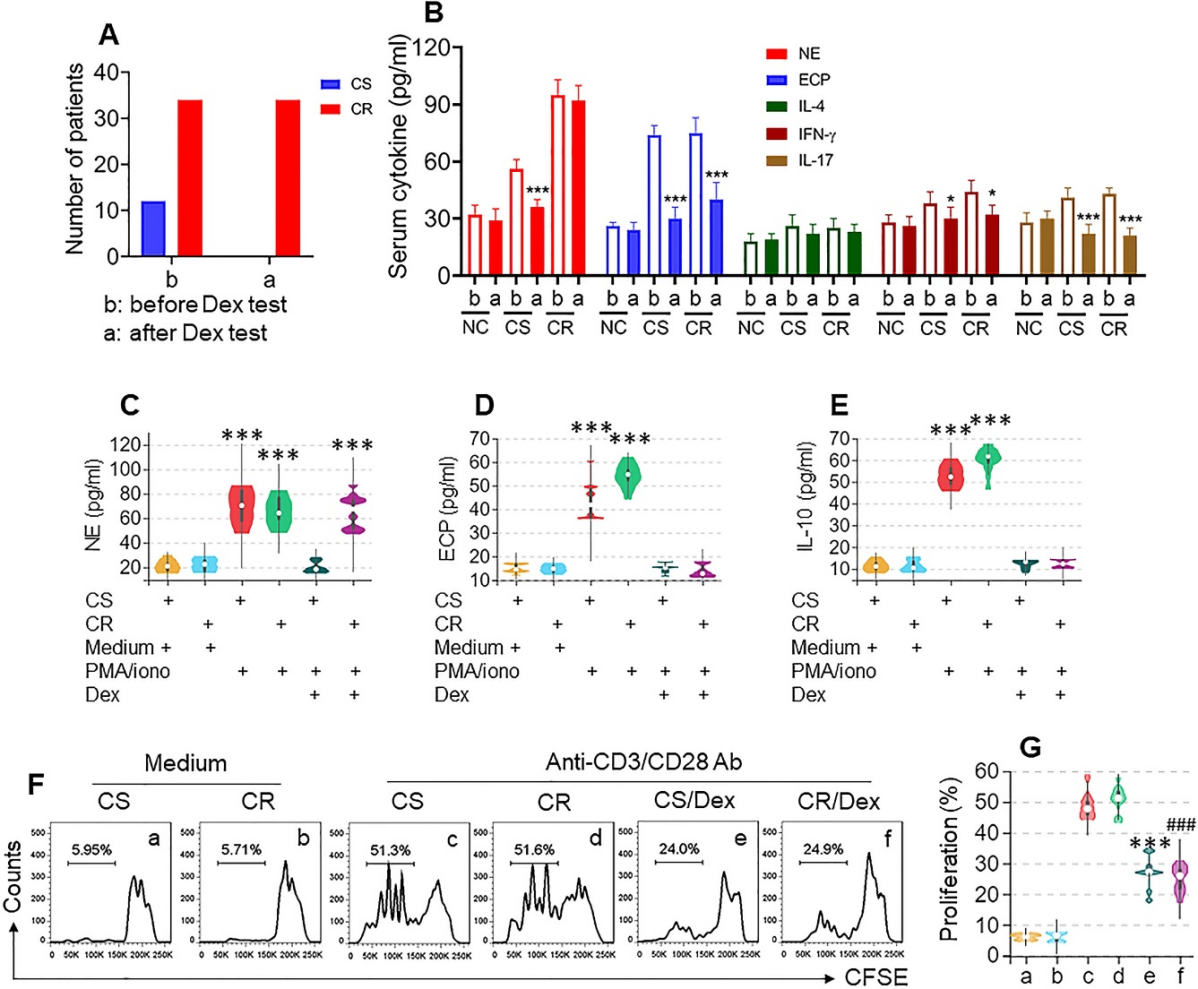
** Assessment of CR and CS status in UC patients**. A-B, CS and CR status of UC patients were determined by the Dex test. A, bars show UC patients with CS (n=12) or CR (n=34) status. B, bars show serum cytokines levels in CS and CR UC patients before and after Dex test. C-G, neutrophils, eosinophils, B cells and T cells were isolated respectively from blood samples of UC patients before Dex test. Neutrophils and eosinophils were stimulated by PMA (50 ng/ml)/iono (ionomycin, 100 ng/ml), B cells were stimulated by LPS (200 ng/ml), with or without the presence of Dex (10^-6^ Mol) in the culture for 48 h. C-E, violin plots show levels of neutrophil elastase (NE, C, from neutrophils), eosinophil cationic protein (ECP, D, from eosinophils) and IL-10 (E, from B cells) in culture supernatant. F, CD3^+^ CD25⁻ T cells (labeled with CFSE) from UC patients were stimulated with anti-CD3 (5 µg/ml) and anti-CD28 (5 µg/ml) Abs in the culture with or without the presence of Dex (10^-6^ Mol) for 3 days. Gated histograms show proliferating T cell frequency. G, violin plots show summarized proliferating T cells. *, p<0.05; ***, p<0.001, compared with group b (B), or CS/medium group (C-E), or group c (G); ###, p<0.001, compared with group d (G). The group labels of panel G are the same as panel F. The data of C-G represent 6 independent experiments.

To determine the CS or CR status of UC patients, the Dex test was carried out. Based on the symptom change after the Dex test, 34 CR patients and 12 CS patients were included in the present study (Figure 1A, Figure S1). The serology test results show that, after the Dex test, neutrophil elastase (NE, a representative neutrophil mediator), eosinophil cationic protein (ECP), IFN-γ, and IL-17 levels were significantly decreased in the CS group, while in the CR group, serum levels of ECP, IFN-γ, and IL-17 were also significantly decreased, but the NE levels were not changed. Serum IL-4 levels were changed in neither the CS group nor CR group (Figure 1B). These results suggest that neutrophils in a portion of UC patients show resistance to Dex.

We further isolated neutrophils, eosinophils, B cells, and T cells from colon samples (Figure S2) of both the CS and CR groups; the cells were cultured separately and activated in the culture. Neutrophils and eosinophils were activated by a non-specific cell activator set, Phorbol 12-myristate 13-acetate (PMA), B cells were stimulated by LPS, and T cells were activated by anti-CD3/CD28 Abs, with or without the presence of Dex for 48 h. Culture supernatants were analyzed by ELISA. We found that, upon activation, the levels of NE in neutrophil culture, ECP in eosinophil culture, and IL-10 in B cell culture were significantly increased, which was abrogated by the presence of Dex in cells obtained from both CS and CR UC patients except the NE levels in CR neutrophil culture, that was almost not changed (Figure 1C-E). CD3^+^ CD25⁻ T cells were stimulated by anti-CD3/CD28 Abs in the culture with or without the presence of Dex. We observed that T cells from both CS and CR groups proliferated drastically in response to the Ab stimulation, which was suppressed by the presence of Dex in both groups (Figure 1F-G). These results indicate that neutrophils of CR UC patients show resistance to Dex, which did not occur in eosinophils, B cells, or T cells.

Histology examination showed neutrophil and eosinophil infiltration in the colon tissue of both CR and CS UC colon tissues (Figure S3A). By fluorescence activating cell sorting (FACS) assay, about 30% of neutrophils were counted in single cells isolated from the lamina propria in the CR group. In comparison, only about 10% of neutrophils were counted in the CS group. Significantly fewer neutrophil and eosinophil counts were detected in the NC group (Figure S3B-E). These results indicate that the CR UC colon tissues have neutrophil-dominant inflammation, suggesting that neutrophils in the CR UC colon tissues may be responsible for the CR status.

### High levels of the TW1 expression are detected in colon tissue neutrophils of CR subjects

**Figure 2:**
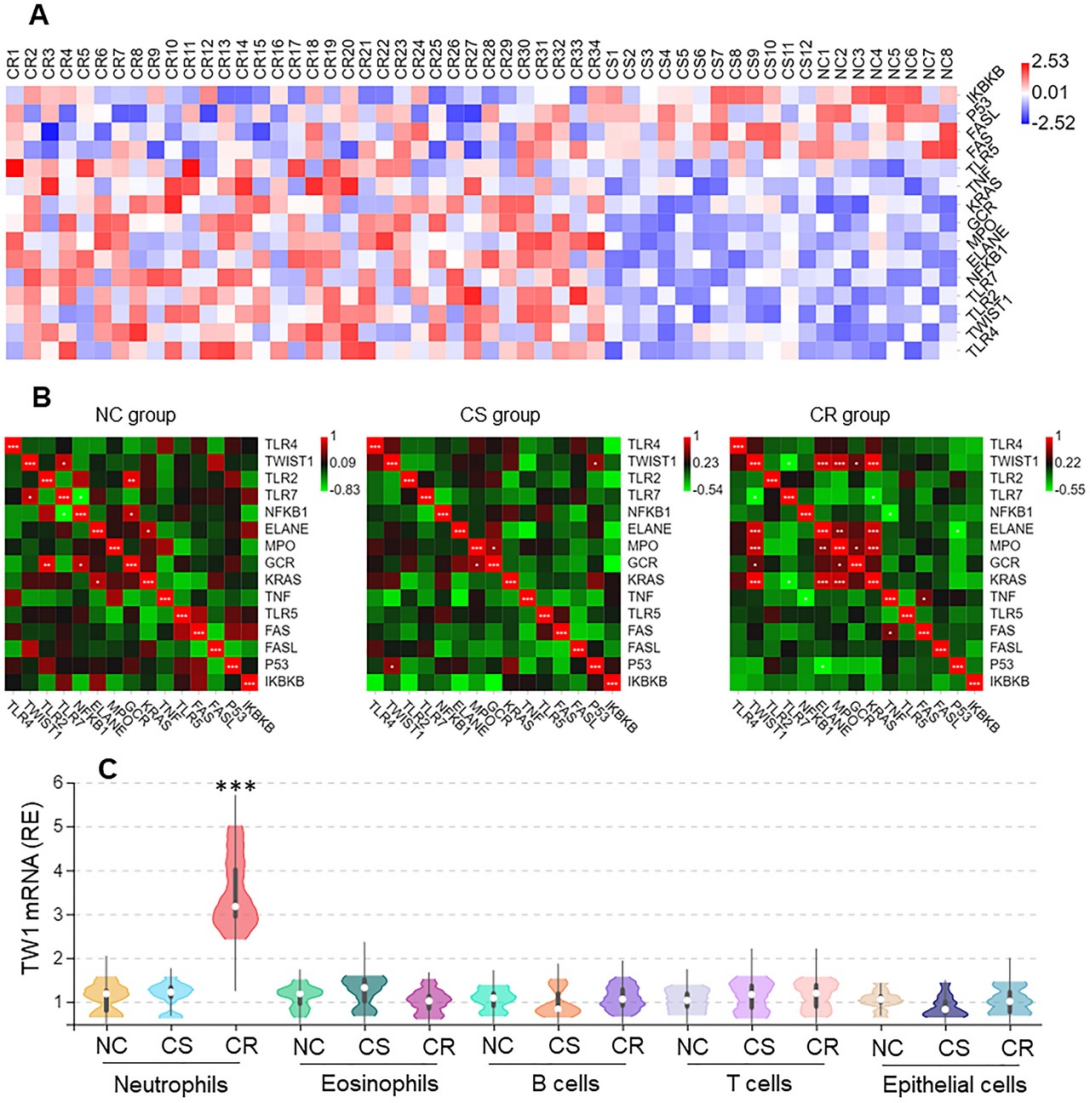
** Higher TW1 and Ras expression in colon tissue neutrophils of UC patients with CR**. Surgically removed colon tissues were obtained from UC patients of CS (n=12) and CR (n=34), and NC subjects (n=8). Neutrophils, eosinophils, B cells, T cells and epithelial cells were isolated from the samples by FACS. A, RNAseq results show 15 DEGs in neutrophils. B, heatmaps show gene expression levels between TW1 and other DEGs. C, RNAs were extracted from isolated immune cells and epithelial cells; TW1 mRNA levels were assessed by conventional RT-qPCR. Violin plots show TW1 mRNA levels. ***, p<0.001 (Mann Whitney test), compared with NC group. The data of violin plots are presented as median, IQR and data range, and represent 6 independent experiments.

Neutrophils from surgically removed colon tissues were analyzed by RNAseq. Among the 15 differentially expressed genes (DEGs), the twist1 (TW1) gene activities were higher in the CR group than in other DEGs (Figure 2A), that was positively correlated Ras, MPO, and ELANE within the CR group, but not in the CS group nor NC group (Figure 2B). The higher expression of TW1 in CR neutrophils was verified by conventional RT-qPCR, but this higher expression was not detected in eosinophils, B cells, or T cells in the CR group, nor in the CS group or NC group (Figure 2C). TW1 was also detectable in colon epithelial cells; its levels were not significantly different between the NC, CR, and CS groups (Figure 2C). These results suggest that TW1 is involved in the CR status of UC patients.

### TW1 interacts with GRα to attenuate the effects of steroids on regulating neutrophil activities

**Figure 3:**
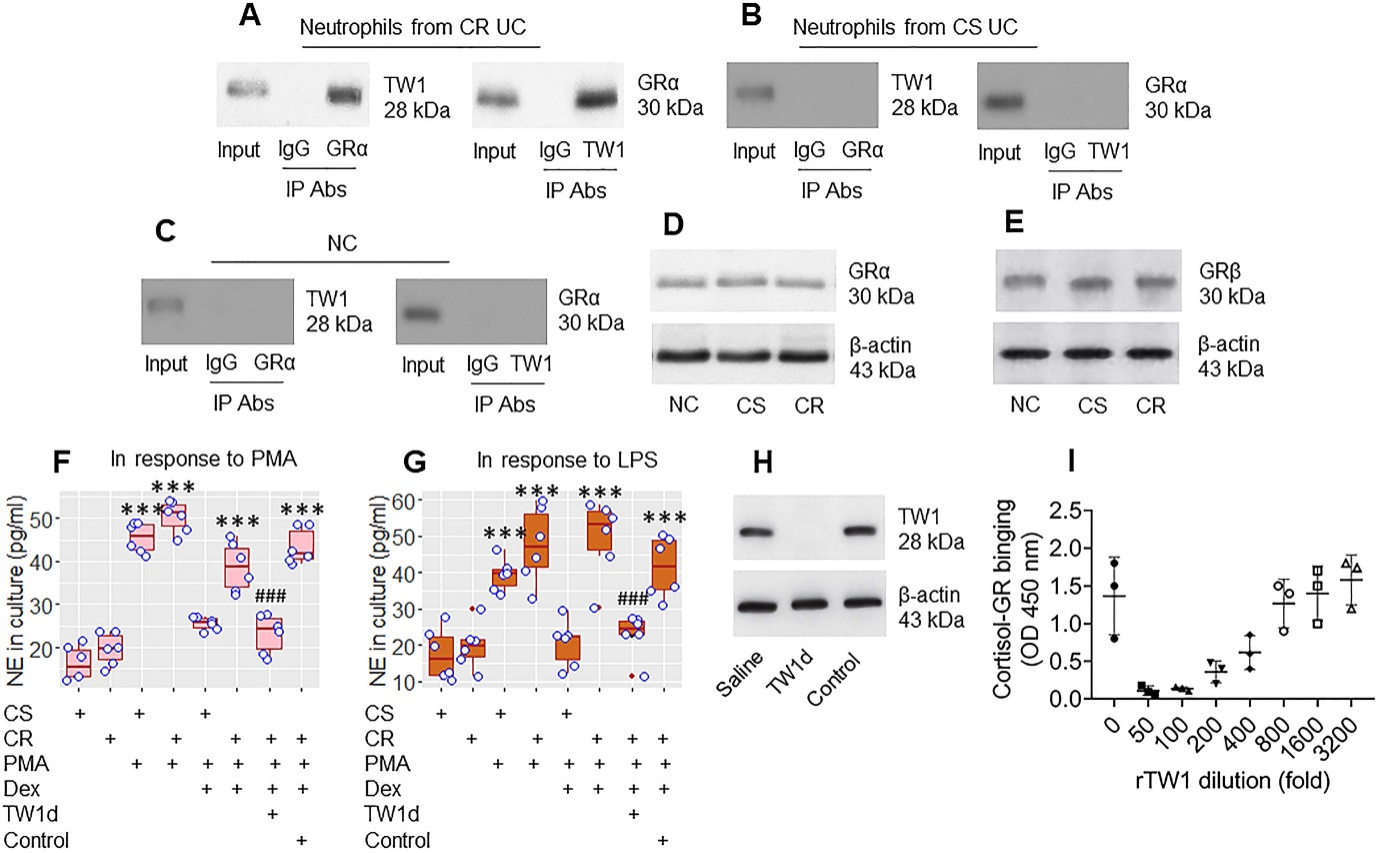
** TW1 interferes with steroid effects on regulating neutrophil activities**. A-C, protein extracts were prepared with colon tissue-isolated neutrophils collected from CR UC patients (n = 19), CS UC patients (n = 12), and NC subjects (n = 8), and analyzed by co-IP with antibodies of GRα or TW1 as the precipitation Ab. The immunoblots show a complex of TW1 and GRα (the data are from one experiment that represent 3 independent experiments with pooled protein samples obtained from all cases per group). D-E, proteins were extracted from neutrophils isolated from surgical colon samples were analyzed by Western blotting. Immunoblots show the GRα (D) and GRβ (E) levels in neutrophils. F-G, isolated colon neutrophils were treated with the agents listed below the bar graph. CS: Corticosteroid sensitive UC patients. CR: Corticosteroid resistant UC patients. PMA: PMA in the culture at 50 nM. LPS: LPS in the culture at 250 ng/ml. Dex: Dexamethasone in the culture at 1 µM. TW1d: TW1-deficient neutrophils (by CRISPR). Control: Neutrophils were treated with control CRISPR reagents. The bars show neutrophil elastase (NE) levels in the culture. H, the immunoblots show TW1 protein in neutrophils (the data are from one experiment that represent 3 independent experiments). I, competitive efficiency of binding to GR between cortisol and TW1. ***, p<0.001 (ANOVA followed by the Dunnett's test), compared with the CS alone group. ###, p<0.001 (the Student *t*-test), compared with the group of neutrophils treated with PMA and Dex. H, The bars show eosinophil cation protein (ECP) levels in the culture supernatant. I, gated FACS plots show apoptotic neutrophils after exposure to PMA in the culture.

The data in Figures 1 and 2 suggest that TW1 in neutrophils plays a role in the CR status of UC patients. To verify this, we prepared protein extracts from colon neutrophils obtained from CR UC patients and CS UC patients and analyzed them by immunoprecipitation with an anti-TW1 Ab as bait. The purpose of this was to detect whether any other proteins bind to TW1; TW1 is a proline-rich protein [it has three PxxP structures in its amino acid sequence at 9-12, 66-69, and 136-139 loci (Figure S4 in supplemental materials) and proline-rich proteins are prone to physically interacting with other proteins 27. The TW1 protein-containing complexes were immunoprecipitated and analyzed by mass spectrometry (MS). The MS results showed that, besides the presence of TW1, the complexes also contained the glucocorticoid receptor (GR)α protein (Figure S5). These results were verified by co-immunoprecipitation (co-IP) (Figure 3A); the TW1/GRα complexes were not detectable in neutrophils collected from the CS group and the NC group (Figure 3B and C), nor in T cells, or B cells, or eosinophils isolated from CR UC colon samples (Figure S6). However, the amounts of GRα and GRβ in neutrophils were not appreciably different between the CR and CS groups (Figure 3D and E). These results suggest that TW1 physically contacts GRα in neutrophils of CR subjects. To elucidate if such a physical contact between TW1 and GRα promotes or attenuates the effects of steroids on neutrophil activities, CR neutrophils and CS neutrophils were prepared and cultured in the presence of phorbol 12-myristate 13-acetate (PMA; a non-specific cell activator) or LPS with or without the presence of dexamethasone (Dex) overnight. We found that exposure to PMA or LPS induced neutrophil elastase (NE, a representative neutrophil mediator, used as an indicator of neutrophil-activating) release, that was attenuated by the presence of Dex in the CS group but not in the CR group. This mirrors the resistance to steroids of CR neutrophils (Figure 3F and G). Depletion of TW1 expression in CR neutrophils by CRISPR (Figure 3H) restored the sensitivity to steroids (Figure 3F and G). Exposure of eosinophils to PMA resulted in ECP release that was efficiently abrogated by the presence of Dex in both the CS and the CR groups (Figure S7). These results demonstrate that TW1 physically contacts GRα in neutrophils, which prevents steroids from binding GRα, and thus, confers neutrophils to the CR property. To verify this, a competitive ELISA was performed. We found that TW1 prevented cortisol from binding glucocorticoid receptors in a dose-dependent manner (Figure 3I). Since activation can induce cell apoptosis, we checked neutrophils by FACS after exposure to PMA or LPS. Less than 2% of neutrophils were apoptotic (Figure S8).

### TW1 is required in the CR development of neutrophils in colitis mice

**Figure 4:**
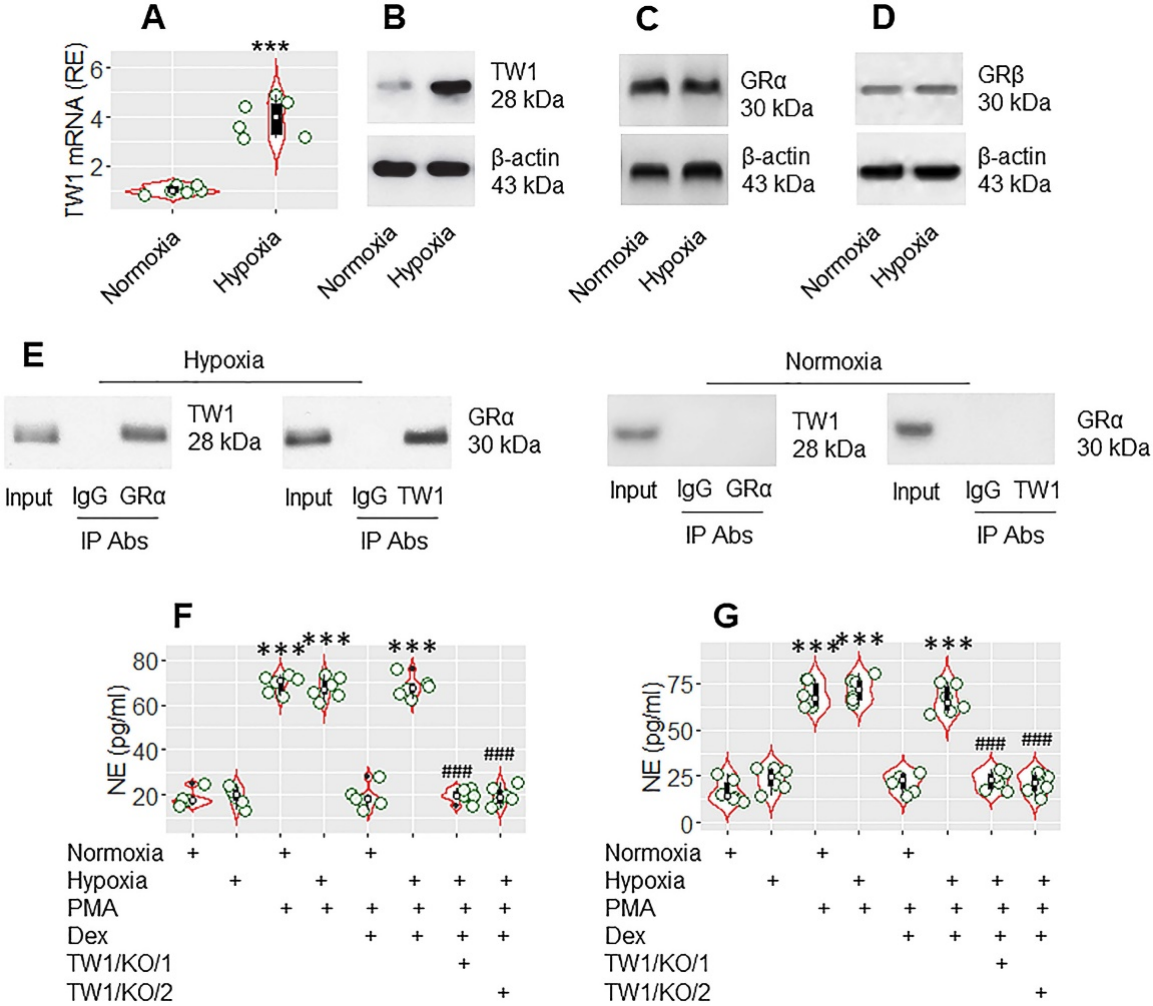
** TW1 is involved in CR development in neutrophils in the mouse colon mucosa**. Mice were treated with hypoxia or normoxia for one week. Neutrophils were isolated from the colon tissues by FACS. A-B, the violin plots show TW1 mRNA levels in neutrophils. The immunoblots show TW1 proteins in neutrophils. C-E, protein extracts of neutrophils were analyzed by Western blotting (C, D) and co-IP (E). Immunoblots show the protein levels of GRα (C), GRβ (D) and complexes of TW1/GRα, but not TW1/GRβ (E). F-G, isolated neutrophils were cultured overnight in the conditions denoted below the violin plots. PMA: PMA in the culture (50 nM). LPS: LPS in the culture (250 ng/ml). Dex: Dex in the culture (1 µM). TW1/KO: Mice with TW1 condition knockout neutrophils. TW1/KO/1: The TW1-knockout was initiated before hypoxia treatment. TW1/KO/2: The TW1-knockout was activated after the hypoxia treatment. The violin plots indicate the NE levels in the culture supernatant. The data of violin plots are presented as median (IQR). Each bubble in violin plots presents data obtained from one experiment. ***, p<0.001, compared with the normoxia group [the Student *t*-test (A) and ANOVA followed by the Dunnett's test (F, G)]. ###, p<0.001, compared with the group treated with hypoxia/PMA/Dex (F), or hypoxia/LPS/Dex (G). The data of B-E are from one experiment, respectively; each of them represents 6 independent experiments.

The data presented Figure 3 suggest that TW1 might be an important factor in CR development. As hypoxia-inducible factor-1α (HIF-1α) promotes TW1 expression 28, mice were treated with hypoxia daily for 1 week following the published procedures 20. Mice were sacrificed on day 8. Neutrophils were isolated from the colon tissues by the enzymatic digestion, followed by FACS-purification. We found that hypoxia treatment markedly increased TW1 expression in neutrophils isolated from the colon tissues (Figure 4A-B), which did not alter the expression of GRα or GRβ in neutrophils (Figure 4C-D). A complex of TW1 and GRα was detected in hypoxia-treated neutrophils (Figure 4E). We then exposed the neutrophils to PMA or PMA and Dex in the culture overnight. As expected, exposure to PMA markedly increased NE release into culture supernatant in both the normoxia group and hypoxia group. The presence of Dex efficiently suppressed NE release in the normoxia group but not in the hypoxia group. Conditional knockdown of the TW1 expression in neutrophils of mice (Figure S9) before hypoxia treatment prevented the CR development in colon tissue neutrophils (Figure 4F; the TW1/KO/1 group). Also, depletion of TW1 expression in neutrophils of mice after hypoxia treatment restored the sensitivity to steroids in neutrophils (Figure 4F; the TW1/KO/2 group). These results demonstrate that TW1 plays a critical role in the CR development in colon tissue neutrophils.

### Ras activation sustains TW1 expression in neutrophils of the colon tissues

**Figure 5:**
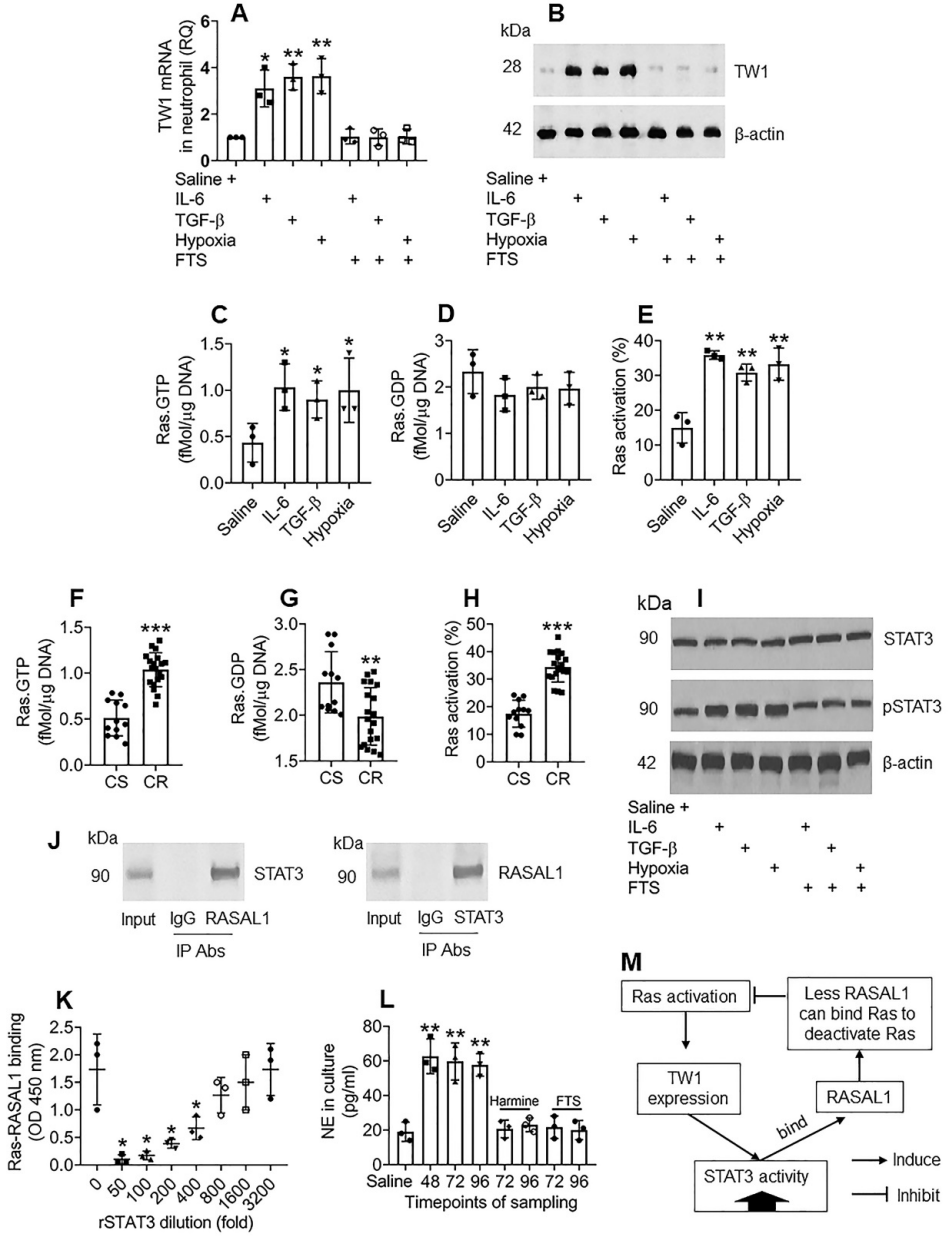
**Ras activation sustains TW1 expression in neutrophils of the colon tissues**. A-E, neutrophils were isolated from blood samples obtained from healthy subjects and cultured with the treatment denoted below the bars of panel A. IL-6: 50 ng/ml. TGF-β: 10 ng/ml. FTS: 75 µM. A, TW1 mRNA levels in neutrophils. B, TW1 protein levels in neutrophils. C-E, bars show levels of Ras.GTPase, Ras.GDPase and Ras activation in neutrophils. F-H, neutrophils were isolated from surgically removed colon tissues of patients with UC and colon cancer, including subjects with corticoid resistance (CR group; n=19), or corticoid sensitiveness (CS group; n=12). The cells were analyzed by Ras-specific ELISA. The bars show levels of Ras.GTPase, Ras.GDPase and Ras activation in neutrophils. I, the immunoblots show STAT3 phosphorylation in naive neutrophils after the treatment denoted below the bars. J, the immunoblots show a protein complex of STAT3/RASAL1 in neutrophils. K, the bars show the binding efficacy of recombinant (r) RASAL1 to rRas after exposing to rSTAT3 at gradient concentrations. L, the bars show NE levels in culture supernatant. M, a flow chart show effect-flow between Ras, TW1 and RASAL1. Harmine: Harmine (a TW1 inhibitor; 10 µM) in the culture. * p<0.05, ** p<0.01, *** p<0.001, compared with the saline group (A, C, D, E, L) or the CS group (F, G, H) or the 0 group (K). Statistical methods: ANOVA followed by the Dunnett's test (A, C, D, E, K, L) or the Mann Whitney test (F, G, H). The data of B, I, J are from one experiment, respectively, that represent 3 independent experiments.

The data in Figures 1-4 demonstrate that TW1 plays a critical role in the CR development of neutrophils in UC colon tissues; thus, it will be important to elucidate the factors sustaining TW1 expression in neutrophils. As shown by the RNAseq results in Figure 2A, Ras gene expression is markedly higher in neutrophils of CR UC patients; we inferred that aberrant Ras activation might be associated with the sustaining status of TW1 expression in CR neutrophils. To test this, we adopted published experimental models of inducing TW1 by exposing naive neutrophils to IL-6, TGF-β, or hypoxia 29-31. In line with those previous reports, treating with either IL-6, TGF-β, or hypoxia, increased TW1 expression as well as high Ras activation in neutrophils, which was abolished by the presence of farnesylthiosalicyclic acid (FTS; a pan-Ras inhibitor) (Figure 5A-E), indicating that Ras activation is required in TW1 expression in neutrophils. Neutrophils isolated from the UC colon mucosa of patients with CR also showed higher Ras activation than those from the CS group (Figure 5F-H). In line with previous reports 32, we found that STAT3 activation was in the signal pathway of TW1 expression upon exposure to either IL-6, TGF-β, or hypoxia (Figure 5I). Therefore, we wondered if STAT3 acts in synergy with Ras activation to sustain the TW1 expression in CR neutrophils since the aberrant Ras activation can sustain cell activities, such as cancer cell proliferation 33. With an anti-STAT3 Ab as a bait, we analyzed protein extracts of colon neutrophils of UC patients with CR by co-IP. The precipitated proteins were analyzed by MS. The MS results revealed that the precipitated proteins contained STAT3 and Ras Protein Activator Like 1 (RASAL1) (Figure S10). We also observed that there were four PxxP structures in the STAT3 amino acid sequences (Figure S11). These results suggest that STAT3 forms a complex with RASAL1. This was verified by co-IP, in which a complex of STAT3/RASAL1 was precipitated using either anti-STAT3 Ab or anti-RASAL1 Ab as the precipitating Ab (Figure 5J). To elucidate whether such a physical contact between STAT3 and RASAL1 interferes with Ras activation, an *in vitro* biochemical assay was performed, in which an ELISA plate was coated with recombinant (r) Ras. Flag-rRASAL1 (that can bind Ras to deactivate Ras) was mixed with rSTAT3 (at gradient concentrations) first, then added to the rRas-coated plate. We found that rRASAL1 could bind rRas, which was attenuated by pre-exposure to rSTAT3 in a dose-dependent manner (Figure 5K). Inhibition of STAT3 attenuated TW1 expression in neutrophils treated with IL-6, TGF-β, or hypoxia (Figure S12). These results suggest that Ras activation induces TW1 expression, during which STAT3 activities are potentiated; STAT3, in turn, potentiates Ras activation by competitively binding RASAL1, and thus, sustaining the TW1 expression in neutrophils. The reasoning was verified by a further experiment, in which exposing neutrophils to hypoxia for 48 h markedly induced NE release into the culture supernatant (Figure 5L). The cells were washed and continued culturing. High levels NE were still detected at 72 h and 96 h, respectively, which was abolished by the presence of harmine (a TW1 inhibitor) or FTS (Figure 5L). The relationship between Ras activation, TW1 expression, and RASAL1 is illustrated in a flow chart (Figure 5M). These results indicate that exposure to hypoxia induces and sustains neutrophil activation that can be inhibited by the TW1 inhibitor or Ras inhibitor.

### Inhibition of TW1 restores CS in colon neutrophils of mice with colitis and CR

**Figure 6:**
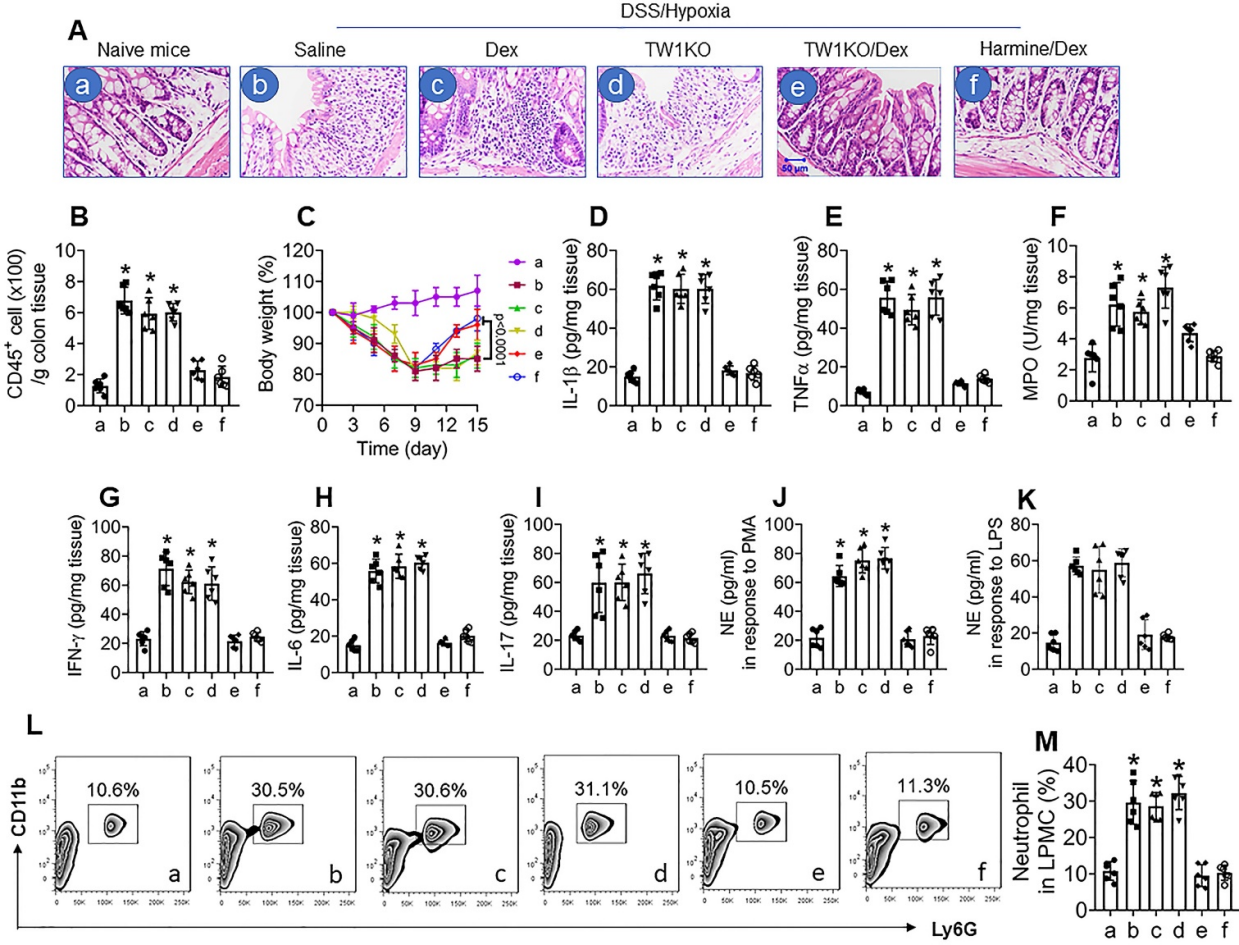
** Inhibition of TW1 restores CS in the colon mucosa**. A murine CR-UC model was developed by treating BALB/c mice with DSS and hypoxia. A, representative histology images show colon tissue structures. DSS/hypoxia: Mice were treated with DSS and hypoxia. Dex: Mice were treated with dexamethasone (5 mg/kg, i.p.) daily for 7 days (day 8-day 14). TW1KO: Mice with TW1-knockout neutrophils. Harmine: Mice were treated with harmine (10 mg/kg, i.p.) daily for 7 days (day 8-day 14). B, the bars show CD45^+^ cell counts (by FACS) in LPMCs isolated from the colon tissues. C, the curves show the body weight changes in the experimental period. D-I, the bars show inflammatory cytokines in the colon tissue protein extracts (by ELISA). J-K, neutrophils were isolated from the colon tissues and stimulated by PMA (J) or LPS (K) in the culture. The bars show NE levels in the culture supernatant. L, the gated FACS plots show neutrophil counts in LPMCs isolated from the colon tissues. M, the bars show summarized neutrophil counts from 6 mice per group. *, p<0.01 (ANOVA followed by the Dunnett's test), compared with group a. The group labels of subpanel B-M are the same as those in subpanel A.

Ultimately, we developed a CR colitis mouse model. After treating with the conventional DSS protocol plus hypoxia, mice showed colitis-like inflammation in the colon, including tissue destruction and profound inflammatory cell infiltration in the colon (Figure 6A and B), bodyweight drop during the experimental period (Figure 6C), increases in proinflammatory cytokines in the colon tissues (Figure 6D-I), increase in neutrophil activities, as shown by the NE-release into culture supernatant in response to PMA- or LPS-stimulation (Figure 6J and K), and increase neutrophil frequency in LPMCs (Figure 6L and M). Exposure to hypoxia alone did not induce inflammation in the colon (Figure S13). Since corticosteroid therapy is the mainstay in the treatment of UC 34, we treated colitis mice with Dex daily for 1 week (from day 8 to day 14). In line with published data 35, treating mice with Dex efficiently suppressed the DSS alone-induced inflammation in the colon (data not shown), but had almost no effects on the DSS-hypoxia-induced colitis in mice (Figure 6A-L), indicating a CR status has been built in the colon by the concurrent exposure to DSS and hypoxia. Based on the data presented in Figures 1-5, the expression of TW1 in neutrophils is responsible for inducing and sustaining the CR status; we inferred the combination of Dex and inhibition of TW1 might be a solution for CR-colitis. To this end, CR-colitis mice were prepared and treated with Dex and harmine (an inhibitor of TW1 36) for 1 week. Indeed, this combination efficiently suppressed the inflammation in the colon (Figure 6). These results also show that depletion of TW1 in neutrophils did not prevent the colitis induction by DSS (Figure S14) or DSS + hypoxia but prevented CR status development (Figure 6). Neutrophils isolated from the colon tissues of DSS + hypoxia-treated wild-type mice, but not those from TW1-depleted neutrophils, showed a CR status. Importantly, we also observed that administration of harmine could restore the sensitivity to steroids in neutrophils, which consequently restored the sensitivity to steroids in the colon (Figure 6). These results demonstrate that TW1 plays a critical role in CR development in the colon. Administration of TW1 inhibitor can restore the sensitivity to steroids in mice with CR-colitis. Additionally, we tested the mechanism by which harmine blocked TW1 in neutrophils. We found that treating CR neutrophils with harmine in the culture did not alter the TW1 mRNA levels but significantly decreased the protein levels in neutrophils, indicating that harmine acted on the post-transcription stage. Further results showed that, by the “peel-re-blotting” approach, ubiquitin was colocalized with TW1 in the samples treated with harmine (Figure S15). These results are in line with previous studies 37, indicating that harmine can promote the degradation of TW1 in neutrophils.

## Discussion

It is the consensus that UC is chronic inflammation in the colon. Current therapies for UC are not satisfactory 3. A large portion of UC patients rely on steroid therapy based on its non-specific suppressive effects on inflammation. However, about 40% of UC patients develop CR status that features inadequate responsiveness or lost sensitivity to the steroid therapy 3. The CR status results in the higher steroid dose requirement and the longer therapeutic period for controlling UC symptoms in the acute flare-up 6. The present study revealed that the higher TW1 expression in neutrophils played a critical role in the CR-status development in the colon; inhibition of TW1 could restore the steroid sensitivity (CS)-status in the colon.

With surgically removed UC colon tissues, we obtained optimal numbers of cellular components to be analyzed. The results showed a high frequency of neutrophils in the colon of UC patients, including both the CS group and the CR group, of which the CR group showed even higher neutrophil counts than that in the CS group. Neutrophils and eosinophils are the inflammatory effector cells in UC 38, among which neutrophils play a more important role in the pathogenesis of UC 1. By invading into the colon tissues, including the lamina propria, crypts, and epithelium, neutrophils release a series of inflammatory mediators, *e.g.*, reactive oxygen species, serine proteases, matrix metalloproteinases, MPO and NE, to degrade tissue structures 1. In line with previous observations 1, the present data show profound neutrophil infiltration in the colon tissues in both the CS group and CR group. Barry et al. also noted that fecal neutrophil elastase (NE) activity reflected disease severity in a IBD patient study and animal model study 39. Although the colon eosinophil counts are more in the UC group than in the NC group, the frequency of eosinophils is significantly less than neutrophils. These data emphasize the important role of neutrophils in UC pathogenesis.

We found that neutrophils from the CR group show CR features that did not respond to the steroid effects, while neutrophils from the CS group responded well to steroids. This provides mechanistic evidence for the clinical phenomenon in UC patients at the CR status. Because neutrophils do not respond to the steroid therapy in CR UC patients, neutrophils may continue releasing inflammatory mediators in response to relevant stimuli, no matter how much steroids are administered to the patients. This reasoning is supported by the laboratory data. The presence of Dex did not suppress the NE release from neutrophils isolated from the CR group patients. The CR status also occurs in asthma patients, in which neutrophils play an important role 40. Eosinophils are also one of the major inflammatory cells in UC, and we did observe more eosinophils in the CS and CR groups than those in the NC group. However, eosinophils isolated from both CS and CR groups responded well to Dex compared with those from the NC group, indicating that eosinophils do not contribute to the CR status within the present cohort of UC patients.

It has been reported that the overexpression of GRβ plays a role in CR development. GRβ can bind the genes of targeting molecules but does not show any regulatory effects on the targets, and thus, interferes with the activities of GRα and the steroid effects 6. Although published data show that the GRβ expression is higher in IBD subject blood cells than in healthy subjects 41, the present data did not show differences in the GRβ expression in colon-isolated neutrophils between the CS group and CR group. The difference between Orii et al. and us might be accounted for by the different samples; Orii et al. observed blood cells, and we observed neutrophils isolated from UC colon tissues. However, our data show higher TW1 expression in neutrophils of the CR group than in the CS group and samples collected from UC/colon cancer patients or UC alone patients. TW1 is an apoptosis inhibitor. Previous studies mainly focused on its effects on cancer cell proliferation and cancer cell metastasis 30-32. The present data revealed another feature of TW1 that was significantly increased in neutrophils of CR colon tissues but not in neutrophils isolated from CS colon tissues or non-UC colon tissues. The present data provide a mechanistic explanation for the CR status in neutrophils isolated from the UC patient colon tissues. TW1 forms a complex with GRα to prevent GRα from binding steroids, and thus, blocks the effects of steroid on suppressing neutrophil activities (*e.g.*, the inflammatory mediator release). Neutrophil-released mediators play canonical roles in acute UC flares 8.

The data show that the TW1 expression in colon neutrophils is generally higher in the CR group. This phenomenon mirrors the sustaining status of TW1 expression. Thus, to elucidate the mechanism by which the TW1 expression remains relatively high is of significance. The data showed that the TW1 expression is positively correlated with the Ras activation. One of the roles of Ras activation is to sustain certain cell activities, *e.g.*, cancer cell proliferation and overgrowth 33. The data implicate that Ras activation is associated with the sustaining TW1 expression in CR neutrophils. The reasoning is supported by the present data; the Ras activation status is higher in CR neutrophils than in CS neutrophils. STAT3, one of the components of the signal pathways of TW1 expression in neutrophils, physically contacts with RASAL1, one of the Ras GTPase activating proteins to prevent the Ras deactivation in neutrophils. From these data, we can envisage a scenario that Ras activation potentiates TW1 expression in neutrophils; TW1, in turn, potentiates Ras activation. This forms a feedback loop to sustain the TW1 expression in neutrophils.

In the clinic, steroids are the most prescribed agents to control clinical symptoms of acute UC flares. In those UC patients with CR status, a higher steroid dosage is required that might result in severe steroid side effects and prolong the therapeutic period to control UC flares 34. The present data demonstrate that depletion of TW1 in neutrophils in colitis mice at CR status can restore the responsiveness to steroid therapy. The combination of steroids and TW1 inhibitor administration efficiently increased the sensitivity to steroids and suppressed the experimental inflammation in the colon of mice at CR status.

In summary, the present data shows that colon neutrophils isolated from UC patients at CR status express higher levels of TW1. TW1 contributes to CR development and maintenance. Inhibition of TW1 can restore sensitivity to steroid therapy in the colon; this has potential for translation to the treatment of UC with CR status.

## Figures and Tables

**Table 1:** Demographic data of human subjects

	CR	CS	NC
Number of patients	19	12	8
Gender (female; %)	11 (57.9)	7 (58.3)	5 (62.5)
Age: M (IQR; y)	45.5 (22-63)	47.2 (28-61)	46.6 (29-58)
Disease duration (y): M (IQR)	7.8 (4-22)	7.2 (3.5-21)	1.5 (0.5-2)
**Disease location: cases (%)**			
*Pan-colitis*	12 (63.2)	8 (66.7)	
*Left sided colitis*	7 (36.8)	4 (33.3)	
partial Mayo score, M (IQR)	7.1 (5.1-7.6)	7.1 (4.6-7.4)	
WBC (/μL; ×1000), M (IQR)	6.3 (5.2-7.5)	6.2 (4.8-7.2)	5.8 (4.4-6.2)
Hemoglobin (g/dL), M (IQR)	8.8 (6.6-11.4)	9.1 (7.2-11.8)	12.5 (11.6-13.5)
Platelet (×10^4^/μL), M (IQR)	26.8 (20.5-32.2)	27.2 (22.5-31.5)	26.6 (21.2-32.2)
Albumin (g/dL), M (IQR)	3.85 (3.4-4.5)	3.96 (3.5-4.4)	3.91 (3.4-4.8)
CRP (mg/dL), M (IQR)	0.25 (0.02-0.85)	0.26(0.03-0.78)	0.26(0.02-0.18)
NLR, M (IQR)	3.25 (1.62-5.28)	3.31(1.85-5.17)	3.29(1.71-5.03)
IL-1β (pg/ml), M (IQR)	104.6(66.6-168.1)*	67.5(45.1-86.9)*^#^	19.7 (13.2-26.5)
TNF-α (pg/ml), M (IQR)	176.5 (65.5-298.4)*	69.4(41.2-88.6)*^#^	11.9 (5.6-31.2)
IL-17 (pg/ml), M (IQR)	56.4 (26.4-77.8)*	61.2 (25.3-92.1)*	15.6 (3.5-33.9)
IL-4 (pg/ml), M (IQR)	25.5 (11.6-35.4)	28.6 (14.4-37.3)	16.5 (11.2-28.8)
**Concomitant medication to corticosteroids**			
Inflaximab (%)	8 (42.1)	5 (41.7)	
Azathioprine (%)	4 (21.1)	2 (16.7)	
6-mercaptopurine (%)	3 (15.8)	1 (8.3)	
Methotrexate (%)	3 (15.8)	2 (16.7)	
Cyclosporine (%)	4 (21.1)	2 (16.7)	
Anti-cancer therapy	0	0	

Abbreviations: CR, corticosteroid resistant group; CS, corticosteroid sensitive group; NC, non-UC control group; M, median; IQR, interquartile range; CRP, C-reactive protein. NLR: neutrophil-to-lymphocyte ratio. *, p<0.01 (Mann Whitney test), compared with the NC group. #, p<0.01 (Mann Whitney test), compared with the CR group.
