# Supplementary material for: Twist1 contributes to developing and sustaining corticosteroid resistance in ulcerative colitis
Source: Theranostics. 2021 Jun 26;11(16):7797–812. doi: 10.7150/thno.62256 (PMC8315068; doi:10.7150/thno.62256)
Supplement: Supplementary file 1 — Supplementary figures and data. [file thnov11p7797s1.pdf]

Supplementary materials

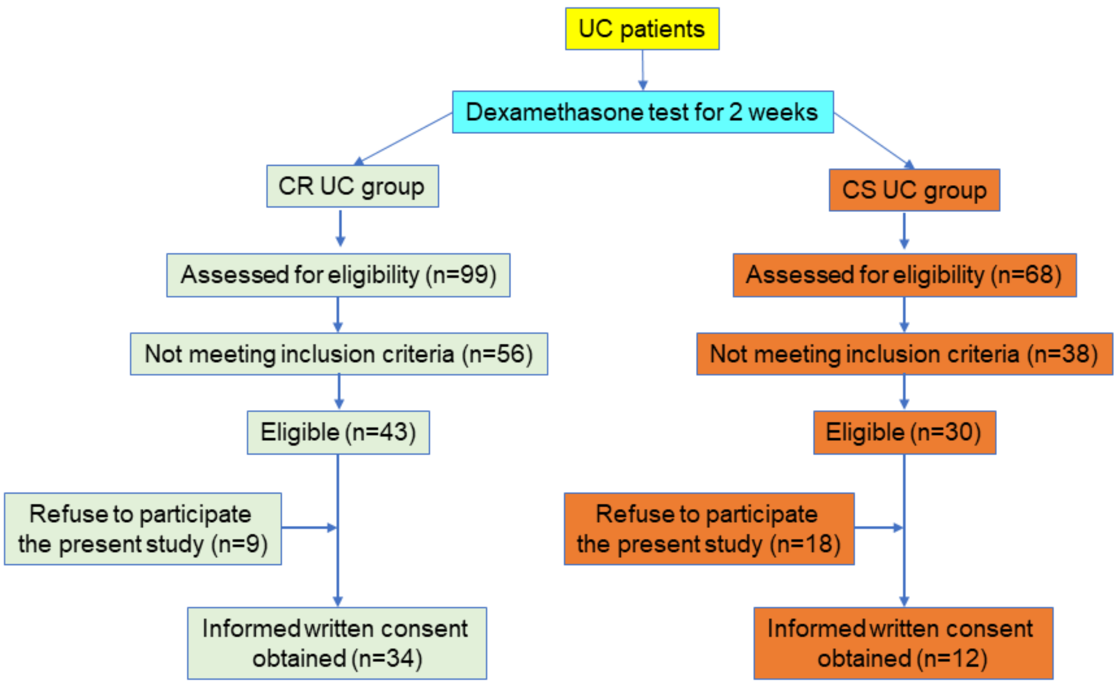

Figure S1. A flow chart shows UC patient selection.

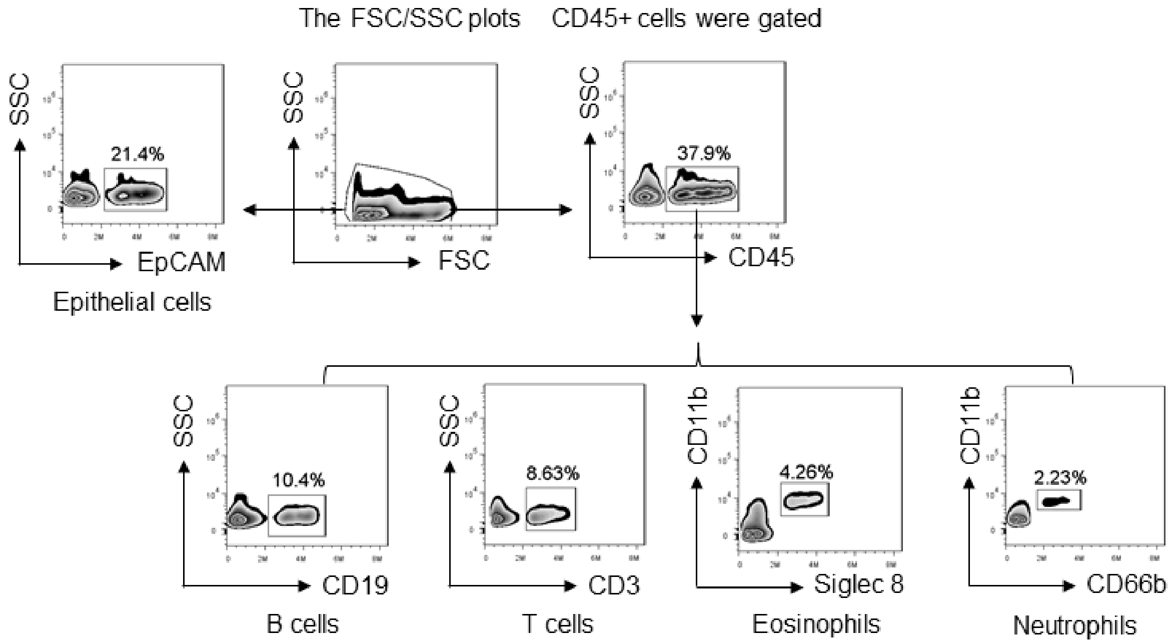

Figure S2. FACS gating procedures. Surgically removed colon tissues from a non-UC patient were used in this experiment to illustrate the FACS gating procedures.

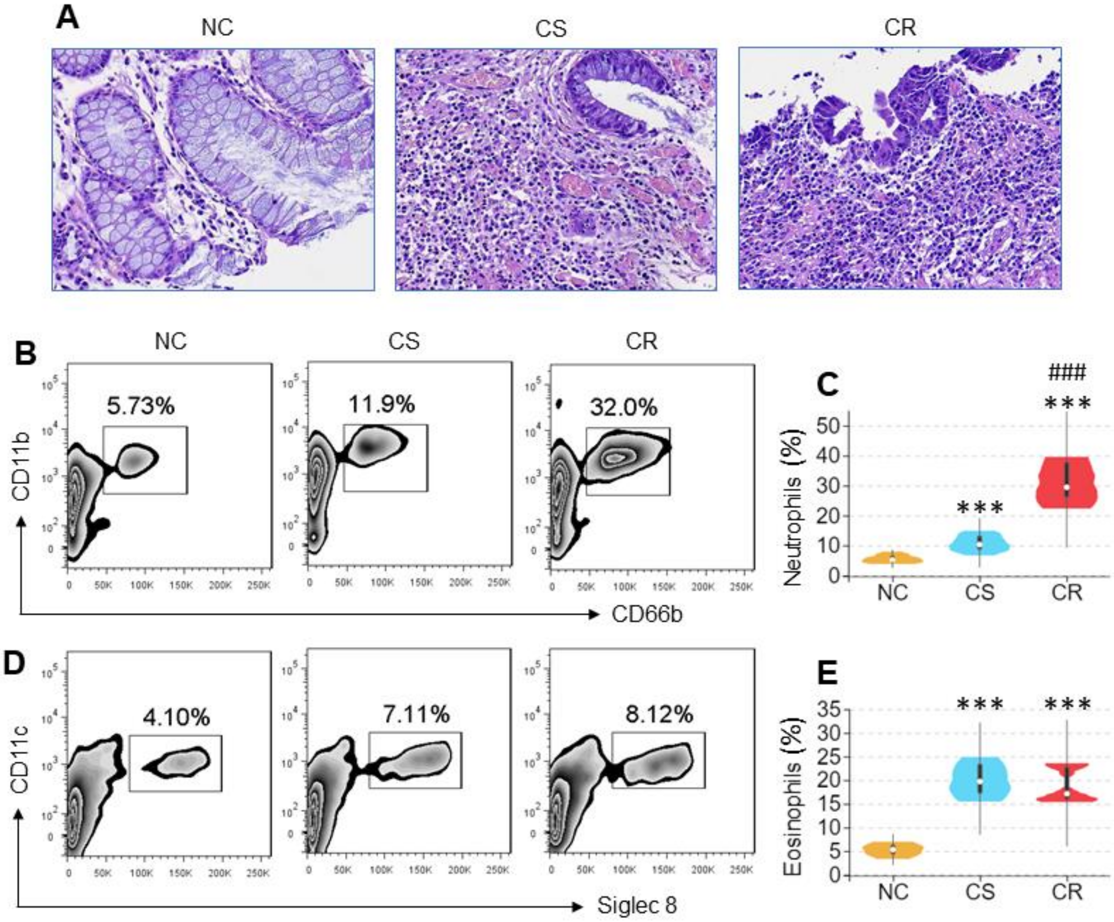

Figure S3. The UC colon tissues of CR patients show high frequency of neutrophils (N). Surgically removed colon tissues were collected from patients with UC and colon cancer, including subjects with corticosteroid resistance (CR group; n=34), or corticosteroid sensitiveness (CS group; n=12). Colon samples from patients with colon cancer without UC were also collected used as non-UC controls (NC; n=8). A, representative colon histology images (original magnification: ×200) show profound inflammatory cell infiltration in the tissues. B-E, lamina propria mononuclear cells (LPMC) were isolated from the colon samples and analyzed by FACS. The gated FACS plots show cell counts of neutrophils (B) and Eo (D). The violin plots show summarized cell counts data of N (C) and Eo (E). ***, $p<0.001$ (Mann Whitney Test) compared with NC group. ###, $p<0.001$, compared with CS group. The data of violin plots are presented as mean (IGR) and data range.

Human TW1 amino acid sequence (NM_000474.4)
MMQDVSSS**PVSP**ADDSLSNSEEEPDRQQPPSGKRGGRKRRSSRRSAGGGAGPGGA
AGGGVGGGDE**PGSP**AQGKRGKKSAGCGGGGGAGGGGGSSSGGGSPQSYEELQTQ
RVMANVRERQRTQSLNEAFAALRKII**PTLP**SDKLSKIQTLKLAARYIDFLYQVLQSDELD
SKMASCSYVAHERLSYAFSVWRMEGAWSMSASH

Figure S4. Three PxxP structures in the amino acid sequence of TW1. Three PxxP structures (in red) in the amino acid sequence of TW1 molecule.

Peptide 1: NLTSLGTLNFPGRTVFSN

Peptide 2: FPQQGQISLSSGETDLK

Human GRα molecular amino acid sequence (GenBank: U01351.1)
MDSKESLTPGREENPSSVLAQERGDVMDFYKTLRGGATVKVSASSPSLAVASQSDSK
QRRLLVDFPKGSVSNAQQPDLSKAVSLSMGLYMGETETKVMGNDLGFPQQGQISLSS
GETDLKLLEESIANLNRSTSVPENPKSSASTAVSAAPTEKEFPKTHSDVSSEQQHLKGQ
TGTNGGNVKLYTTDQSTFDILQDLEFSSGSPGKETNESPWRSDLL
IDENCLLSPLAGEDDSFLLEGNSNEDCKPLILPDTKPKIKDNGDLVLSSPSNVTLPQVKT
EKEDFIELCTPGVIKQEKLGTVYCQASFPGANIIGNKMSAISVHGVSTSGGQMYHYDMN
TASLSQQQDQKPIFNVIPPIPVGSENWNRCQGSGDDNLTSLGTLNFPGRTVFSNGYSS
PSMRPDVSSPPSSSSTATTGPPPKLCLVCSDEASGCHYGVLTCGSCKVFFKRAVEGR
QHNYLCAGRNDCIIDKIRRKNCPACRYRKCLQAGMNLEARKTKKKIKGIQQATTGVSQE
TSENPGNKTIVPATLPQLTPTLVSLLEVIEPEVLYAGYDSSVPDSTWRIMTTLNMLGGRQ
VIAAVKWAKAIPGFRNLHLDDQMTLLQYSWMFLMAFALGWRSYRQSSANLLCFAPDLII
NEQRMTLPCMYDQCKHMLYVSSELHRLQVSYEEYLCMKTLLLLSSVPKDGLKSQELF
DEIRMTYIKELGKAIVKREGNSSQNWQRFYQLTKLLDSMHEVVENLLNYCFQTFLDKTM
SIEFPEMLAEIITNQIPKYSNGNIKKLLFHQK

Figure S5. Identification of GRα and in a complex precipitated by an anti-TW1 Ab. A complex in protein extracts of neutrophils isolated from UC colon tissues of patients with CR was analyzed by MS. The two representative peptides indicate that they belong to GRα molecule.

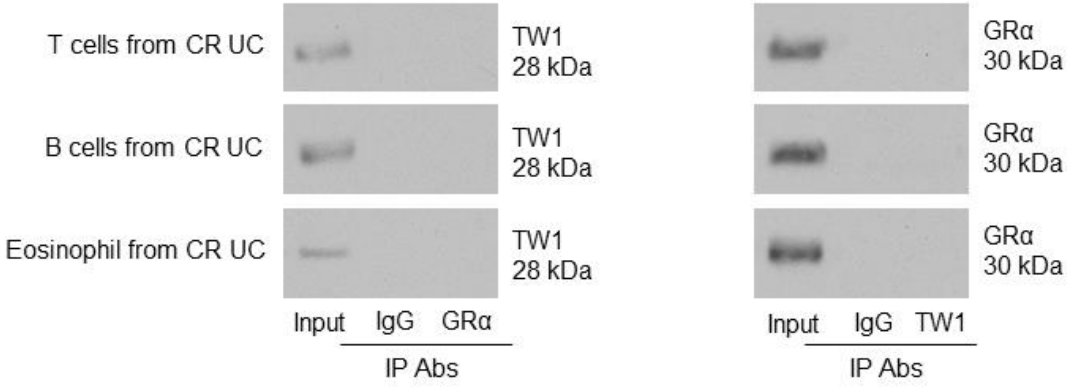

Figure S6. Assessment of TW1/GRα complex in immune cells isolated from CR UC colon tissues. T cells, B cells and eosinophils were isolated from CR UC colon tissues as described and analyzed by co-IP with anti-TW1 Ab and GRα Ab as precipitating Abs and blotting Abs, respectively. Immunoblots show co-IP results. The data are from one experiment that represent 6 independent experiments.

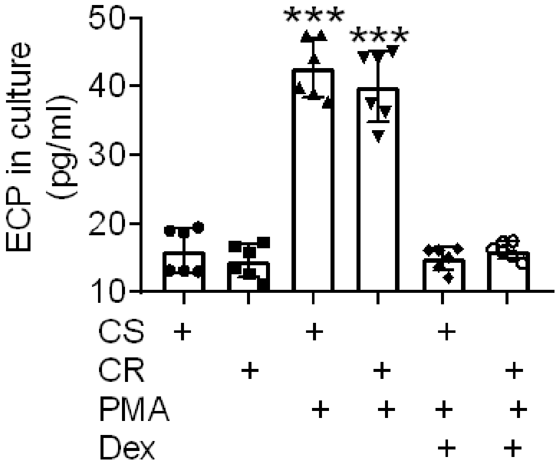

Figure S7. Eosinophils isolated from CR and CS colon tissues do not show CR. Isolated colon eosinophils were treated with the agents listed below the bar graph. CS: Corticosteroid sensitive UC patients. CR: Corticosteroid resistant UC patients. PMA: PMA in the culture at 50 nM. Dex: Dexamethasone in the culture at 1 µM. The bars show ECP levels in the culture. ***, p<0.001 (ANOVA followed by Dunnectt's test), compared with CS alone group.

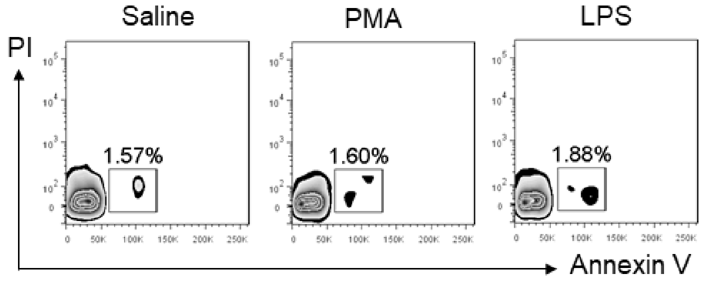

Figure S8. Assessment of neutrophil apoptosis. Isolated CR neutrophils were cultured in the presence of PMA (50 ng/ml) or LPS (250 ng/ml) overnight. The cells were stained with Annexin V reagent and PI, and analyzed by FACS. The gated FACS plots show apoptotic cells. The data represent 3 independent experiments.

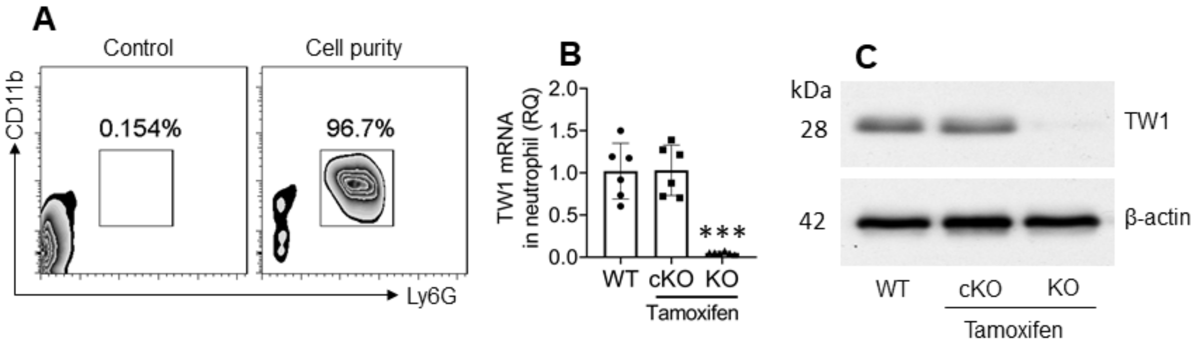

Figure S9. Checking the TW1 expression in neutrophils of mice carrying the TW1-knockout neutrophils. Neutrophils were isolated from the colon tissues of WT mice, mice carrying neutrophils have a WT TW1 gene and a Cre gene (cKO), and mice carrying the TW1-knockout neutrophils (KO) by FACS. A, the gated plots show cell purity of isolated neutrophils. B, the bars show TW1 mRNA levels in neutrophils. C, the immunoblots show TW1 protein levels in neutrophils. Tamoxifen: Mice were treated with tamoxifen daily for one week to activate the TW1-gene-depletion.

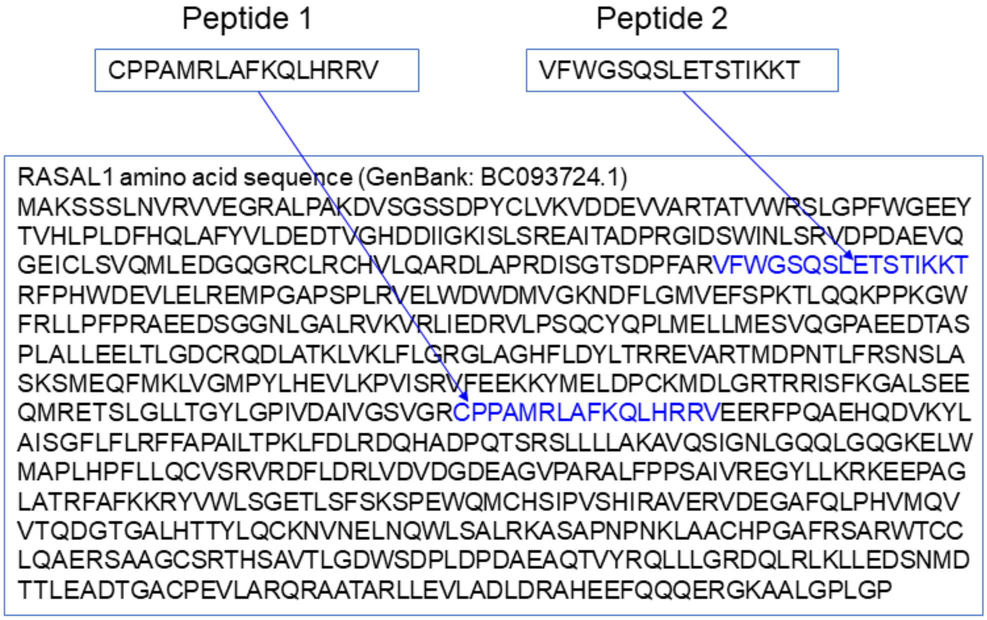

Figure S10. Identification of proteins bound to STAT3 in neutrophils. Proteins were extracted from neutrophils isolated from the colon tissues of UC patients with CR and analyzed by MS. The two representative peptides (the arrows point the original sites; in blue) indicate the protein bound STAT3 is RASAL1L.

Human STAT3) amino acid sequence (NM_139276.3)
MAQWNQLQQLDTRYLEQLHQLYSDSFPMELRQFLAPWIESQDWAYAASKESHATLVFHNLL
GEIDQQYSRFLQESNVLYQHNLRRIKQFLQSRYLEKPMEIARIVARCLWEESRLLQTAATAAQ
QGGQANHPTAAVVTEKQQMLEQHLQDVRKRVQDLEQKMKVVENLQDDFDFNYKTLKSQG
DMQDLNGNNQSVTRQKMQQLEQMLTALDQMRRSIVSELAGLLSAMEYVQKTLTDEELADW
KRRQQIACIGGPPNICLDRLENWITSLAESQLQTRQQIKKLEELQQKVSYKGDPIVQHRPMLE
ERIVELFRNLMKSAFVVERQ**PCMPMHP**DRPLVIKTGVQFTTKVRLLVKFPELNYQLKIKVCID
KDSGDVAALRGSRKFNILGTNTKVMNMEESNNGSLSAEFKHLTLREQRCGNGGRANCDAS
LIVTEELHLITFETEVYHQGLKIDLETHSLPVVVISNICQMPNAWASILWYNMLTNNPKNVNFFT
KPPIGTWDQVAEVLSWQFSSTTKRGLSIEQLTTLAEKLLGPGVNYSGCQITWAKFCKENMAG
KGFSFWVWLDNIIDLVKKYILALWNEGYIMGFISKERERAILSTKPPGTFLLRFSESSKEGGVT
FTWVEKDISGKTQIQSVEPYTKQQLNNMSFAEIIMGYKIMDATNILVSPLVYLY**PDIP**KEEAFGK
YCRPESQEHPEADPGSAAPYLKTKFICVTPTTCSNTIDL**PMSP**RTLDSLMQFGNNGEGAEPS
AGGQFESLTFDMELTSECATSPM

Figure S11. PxxP structures in STAT3 amino acid sequence. There are four “PxxP” structures (in red) in the STAT3 amino acid sequence.

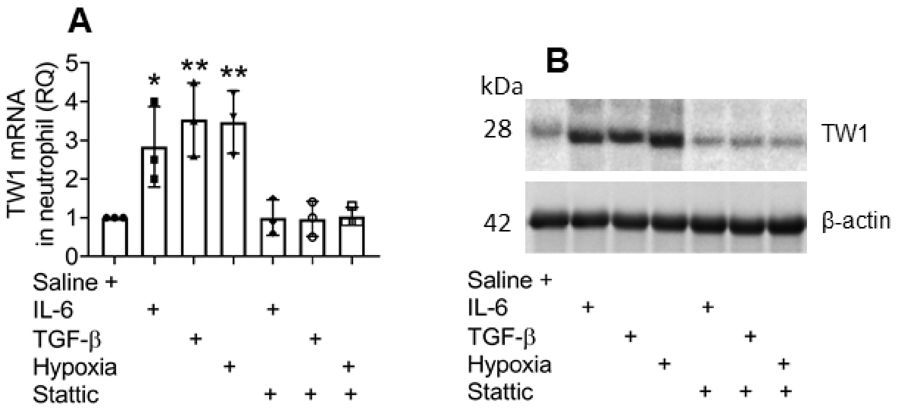

Figure S12. Inhibition of STAT3 attenuates TW1 expression in neutrophils. Neutrophils were isolated from blood samples obtained from healthy human subjects and exposed to IL-6 (50 ng/ml) or TGF-β (10 ng/ml) in the culture, or treated with hypoxia for 24 h. Stattic (an inhibitor of STAT3; 1 μM) was added to the culture in separate wells of each experimental setting. A, the bars indicate TW1 mRNA levels in neutrophils. B, the immunoblots indicate TW1 protein levels in neutrophils. *, $p<0.01$ (ANOVA followed by the Dunnett’s test), compared with the saline group.

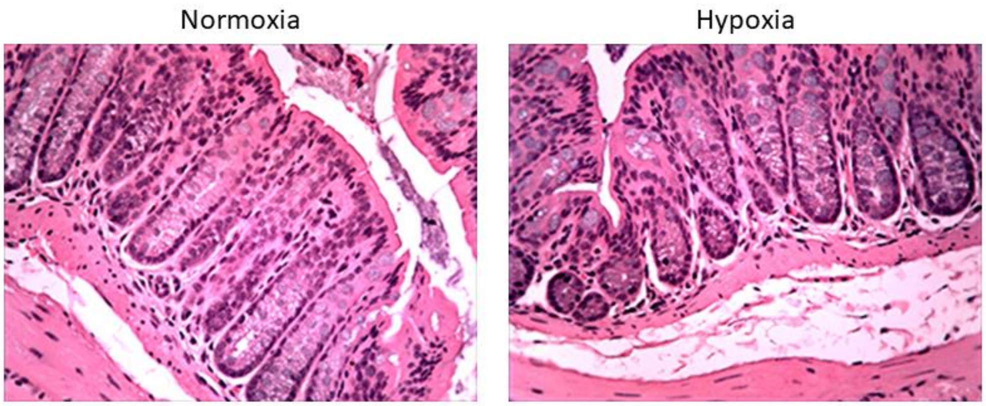

Figure S13. Colon histology of mice treated with normoxia or hypoxia. Used as controls, naïve BALB/c mice were treated with normoxia alone or hypoxia alone daily for one week. Colon segments were excised and processed for paraffin section and HE staining. The representative images show colon tissue structure. Original magnification: ×200.

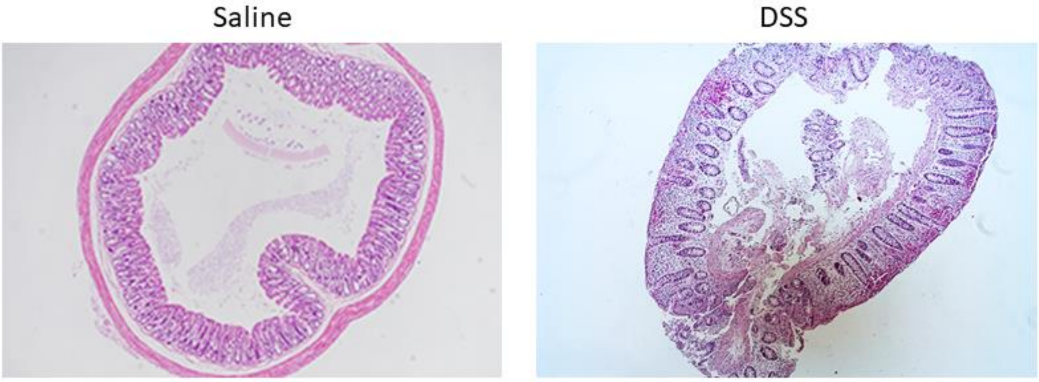

Figure S14. Colon histology of mice carrying TW1-deficient neutrophils. Mice carrying TW1-deficient neutrophils were treated with saline or DSS in the same procedures as described in Fig. 6. Representative images show histology of the colon. Original magnification: ×50. Each group consists of 6 mice.

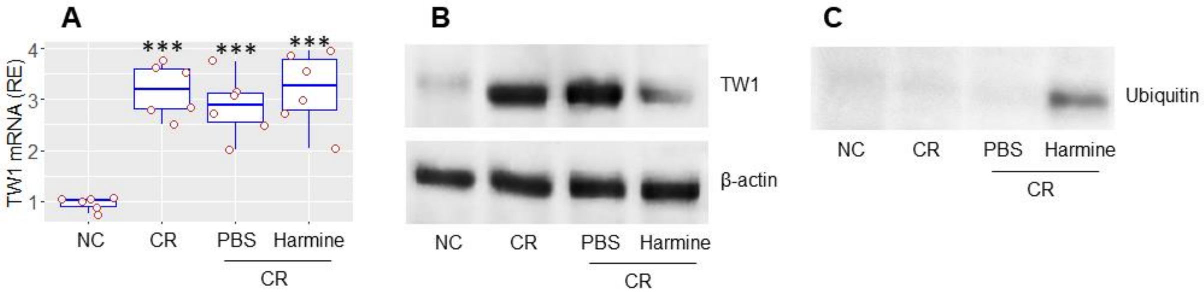

Figure S15. Assessment of the role of harmine in regulating TW1 in neutrophils. Neutrophils were isolated from surgically removed colon tissues of NC (n=6) subjects and CR/UC subjects (n=6), and cultured with the conditions denoted below the graphs for 48 h. Neutrophils were

harvested at the end of culture and analyzed by RT-qPCR and Western blotting. A, boxplots show TW1 mRNA levels in neutrophils. B, immunoblots show TW1 protein levels in neutrophils. C, the PVDF membrane of TW1 blots was processed with the “peel-re-blotting” procedures, and re-blotted with anti-ubiquitin Ab. Immunoblots show ubiquitin levels. Each bubble in boxplots presents data obtained from one sample. ***, $p<0.001$ (ANOVA + Dunnett’s test), compared with the NC group. The data of panel B and C are from one experiment that represent 6 independent experiments. Harmine = 20 μM.

RNAseq data

1	Case	CR1
2	TLR4	-0.68712598
3	TWIST1	-0.44358750
4	TLR2	1.18547924
5	TLR7	1.28878456
6	NFKB1	-0.71137628
7	ELANE	2.966901374
8	MPO	2.72582631
9	GCR	1.01989631
10	KRAS	0.38960035
11	TNF	0.11251925
12	TLR5	3.12821995
13	FAS	0.07774891
14	FASL	0.26436271
15	P53	0.71148680
16	IKBKB	-0.863841577
17	Case	CR2
18	TLR4	1.96417744
19	TWIST1	1.79655633
20	TLR2	2.12619300
21	TLR7	2.01449800
22	NFKB1	3.36321229
23	ELANE	2.680436516
24	MPO	0.03591331
25	GCR	2.95792496
26	KRAS	2.05092523
27	TNF	1.08278887
28	TLR5	0.90102395
29	FAS	-1.38801371
30	FASL	-0.06661543
31	P53	-1.27696332
32	IKBKB	0.670539501
33	Case	CR3
34	TLR4	0.75243616
35	TWIST1	2.32983646
36	TLR2	0.16422712
37	TLR7	0.50364018
38	NFKB1	2.06068094
39	ELANE	0.196005959
40	MPO	-0.10823946
41	GCR	-0.96559534
42	KRAS	-0.64092700

43	TNF	3.39869110
44	TLR5	1.41860851
45	FAS	-1.78160428
46	FASL	-2.15011975
47	P53	0.52894928
48	IKBKB	0.304580800
49	Case	CR4
50	TLR4	3.23968781
51	TWIST1	0.93991106
52	TLR2	-0.91017805
53	TLR7	2.40630034
54	NFKB1	3.13943856
55	ELANE	2.489153628
56	MPO	1.59584361
57	GCR	3.44498788
58	KRAS	0.03510055
59	TNF	0.30500205
60	TLR5	0.22565391
61	FAS	-0.58530470
62	FASL	-0.91984948
63	P53	-1.64206644
64	IKBKB	0.373168037
65	Case	CR5
66	TLR4	-0.67244489
67	TWIST1	-0.93259568
68	TLR2	-0.72375832
69	TLR7	-0.86158416
70	NFKB1	2.38278776
71	ELANE	3.226432689
72	MPO	0.36675346
73	GCR	0.06210111
74	KRAS	0.91110407
75	TNF	0.88982297
76	TLR5	2.48911739
77	FAS	-1.21013470
78	FASL	-0.34537670
79	P53	0.01505806
80	IKBKB	-0.081377947
81	Case	CR6
82	TLR4	-0.80485561
83	TWIST1	1.88880814
84	TLR2	2.46150484
85	TLR7	1.53931365
86	NFKB1	-0.95307987

87	ELANE	1.214824854
88	MPO	3.00803985
89	GCR	3.43147746
90	KRAS	1.90798415
91	TNF	-0.87124553
92	TLR5	0.78964649
93	FAS	-1.15156574
94	FASL	-1.46349254
95	P53	-0.08834014
96	IKBKB	-1.083621462
97	Case	CR7
98	TLR4	1.88247768
99	TWIST1	2.43708237
100	TLR2	0.20322915
101	TLR7	-0.36005489
102	NFKB1	2.24226316
103	ELANE	2.252873896
104	MPO	2.23919411
105	GCR	3.26004675
106	KRAS	0.08919236
107	TNF	0.68460297
108	TLR5	0.99656275
109	FAS	0.49119414
110	FASL	-0.09410940
111	P53	-1.75859842
112	IKBKB	-0.943420631
113	Case	CR8
114	TLR4	1.09053346
115	TWIST1	3.33483878
116	TLR2	2.62525250
117	TLR7	1.60018059
118	NFKB1	2.64057963
119	ELANE	-0.886840609
120	MPO	-0.84456990
121	GCR	0.99573604
122	KRAS	2.25995849
123	TNF	0.36978208
124	TLR5	-0.12264764
125	FAS	0.18507487
126	FASL	-0.72364098
127	P53	-1.73324643
128	IKBKB	-1.020138860
129	Case	CR9
130	TLR4	0.00972133

131	TWIST1	0.97719498
132	TLR2	-0.15677576
133	TLR7	-0.15071077
134	NFKB1	3.42055053
135	ELANE	-0.292642005
136	MPO	-0.94167255
137	GCR	0.80141348
138	KRAS	1.68318163
139	TNF	0.24468744
140	TLR5	1.01279500
141	FAS	-1.99218211
142	FASL	-0.09291155
143	P53	-0.45435117
144	IKBKB	0.395139195
145	Case	CR10
146	TLR4	2.80096262
147	TWIST1	0.01810020
148	TLR2	2.86940298
149	TLR7	-0.39532259
150	NFKB1	3.13087548
151	ELANE	0.346938565
152	MPO	-0.44238651
153	GCR	1.21930273
154	KRAS	3.22735089
155	TNF	3.04152887
156	TLR5	-0.58182140
157	FAS	-0.35246497
158	FASL	-1.20150465
159	P53	-1.00478373
160	IKBKB	-0.991862912
161	Case	CR11
162	TLR4	-0.01839248
163	TWIST1	0.22089296
164	TLR2	2.74574465
165	TLR7	1.19257888
166	NFKB1	0.29899553
167	ELANE	3.011491743
168	MPO	-0.66524229
169	GCR	-0.48368294
170	KRAS	0.33213961
171	TNF	2.82252641
172	TLR5	3.05409071
173	FAS	-1.64035245
174	FASL	-0.50455701

175	P53	0.57339373
176	IKBKB	-1.359810982
177	Case	CR12
178	TLR4	3.27624856
179	TWIST1	1.26713378
180	TLR2	0.23811773
181	TLR7	2.15955748
182	NFKB1	0.32513710
183	ELANE	-0.526603559
184	MPO	-0.21071366
185	GCR	2.58026023
186	KRAS	-0.30853134
187	TNF	0.59406187
188	TLR5	-0.82357393
189	FAS	-1.24853807
190	FASL	-0.43198466
191	P53	-1.95412661
192	IKBKB	0.853557340
193	Case	CR13
194	TLR4	3.45520334
195	TWIST1	1.21778646
196	TLR2	0.83674539
197	TLR7	1.21117514
198	NFKB1	3.20305855
199	ELANE	1.081967814
200	MPO	2.62815480
201	GCR	3.37284866
202	KRAS	-0.28899426
203	TNF	1.88995414
204	TLR5	0.47020548
205	FAS	-0.15723199
206	FASL	0.13191799
207	P53	0.53372144
208	IKBKB	-2.008463214
209	Case	CR14
210	TLR4	1.91341583
211	TWIST1	0.52943830
212	TLR2	2.03965265
213	TLR7	0.15812619
214	NFKB1	2.82385554
215	ELANE	0.026559046
216	MPO	1.40087041
217	GCR	3.22207976
218	KRAS	2.03658592

219	TNF	3.26269455
220	TLR5	1.79557459
221	FAS	-0.14368108
222	FASL	-1.11657175
223	P53	-0.91275597
224	IKBKB	-1.992053136
225	Case	CR15
226	TLR4	0.54958870
227	TWIST1	-0.80330529
228	TLR2	2.38688398
229	TLR7	1.65832838
230	NFKB1	0.63322481
231	ELANE	2.515360357
232	MPO	-0.70553272
233	GCR	0.90326361
234	KRAS	2.42229956
235	TNF	-0.82790819
236	TLR5	0.60541421
237	FAS	-1.01675669
238	FASL	-1.72678406
239	P53	0.20280645
240	IKBKB	-1.687887278
241	Case	CR16
242	TLR4	2.23291891
243	TWIST1	0.27972299
244	TLR2	0.51151698
245	TLR7	-0.16733926
246	NFKB1	-0.58707465
247	ELANE	-0.968132430
248	MPO	-0.20771790
249	GCR	0.36406835
250	KRAS	1.22420361
251	TNF	1.24270623
252	TLR5	-0.39760139
253	FAS	-1.15522813
254	FASL	-0.79653425
255	P53	-1.28711329
256	IKBKB	-0.049264747
257	Case	CR17
258	TLR4	-0.81055818
259	TWIST1	2.11967163
260	TLR2	0.63586881
261	TLR7	-0.76087055
262	NFKB1	-0.65521876

263	ELANE	3.407629768
264	MPO	1.63259324
265	GCR	2.49832097
266	KRAS	0.46672765
267	TNF	-0.96081949
268	TLR5	2.35634451
269	FAS	-0.56210378
270	FASL	-0.41772743
271	P53	0.21886435
272	IKBKB	-1.712531417
273	Case	CR18
274	TLR4	2.40664228
275	TWIST1	3.00939059
276	TLR2	0.95032380
277	TLR7	1.10829973
278	NFKB1	2.62037729
279	ELANE	3.159843525
280	MPO	1.50079627
281	GCR	0.14385890
282	KRAS	0.50589773
283	TNF	3.34032753
284	TLR5	-0.75849825
285	FAS	0.59448857
286	FASL	0.83931024
287	P53	-0.64145117
288	IKBKB	0.516809554
289	Case	CR19
290	TLR4	0.02300301
291	TWIST1	1.88838167
292	TLR2	2.86755592
293	TLR7	2.42320956
294	NFKB1	-0.14120036
295	ELANE	2.854303601
296	MPO	1.48468751
297	GCR	-0.82986278
298	KRAS	1.86274315
299	TNF	2.32790793
300	TLR5	2.19471432
301	FAS	-1.03428833
302	FASL	-0.90597349
303	P53	0.15396124
304	IKBKB	-1.241465139
305	Case	CR20
306	TLR4	3.22579295

307	TWIST1	3.14529466
308	TLR2	2.42534689
309	TLR7	0.48155525
310	NFKB1	-0.27176388
311	ELANE	-0.090360338
312	MPO	-0.74294479
313	GCR	0.27533842
314	KRAS	-0.99670996
315	TNF	3.42510267
316	TLR5	1.30476016
317	FAS	-1.98842256
318	FASL	0.05550583
319	P53	0.50884373
320	IKBKB	0.268955714
321	Case	CR21
322	TLR4	3.38301394
323	TWIST1	-0.46771070
324	TLR2	2.50868765
325	TLR7	2.59709646
326	NFKB1	2.44865303
327	ELANE	0.535045684
328	MPO	2.26199530
329	GCR	2.71629399
330	KRAS	-0.65813105
331	TNF	-0.51184676
332	TLR5	0.89945264
333	FAS	0.52970454
334	FASL	-0.41153632
335	P53	-0.62168605
336	IKBKB	-1.828640169
337	Case	CR22
338	TLR4	0.27502028
339	TWIST1	-0.88950103
340	TLR2	1.26486873
341	TLR7	-0.40870506
342	NFKB1	-0.32287673
343	ELANE	-0.243855836
344	MPO	3.30233462
345	GCR	2.42330808
346	KRAS	-0.68327495
347	TNF	0.98237843
348	TLR5	1.72484135
349	FAS	-0.34877614
350	FASL	-0.87878743

351	P53	0.54277769
352	IKBKB	-1.803161503
353	Case	CR23
354	TLR4	1.01344842
355	TWIST1	-0.96108296
356	TLR2	0.91728069
357	TLR7	-0.97753115
358	NFKB1	2.90912036
359	ELANE	2.359157233
360	MPO	1.49737065
361	GCR	3.27190154
362	KRAS	1.56634159
363	TNF	0.25528179
364	TLR5	1.71635660
365	FAS	0.92514166
366	FASL	0.54578311
367	P53	0.39042885
368	IKBKB	-0.678267577
369	Case	CR24
370	TLR4	0.22905628
371	TWIST1	0.74586539
372	TLR2	-0.52328368
373	TLR7	1.61535234
374	NFKB1	0.77452040
375	ELANE	0.584378020
376	MPO	0.16812264
377	GCR	0.19068047
378	KRAS	1.81062305
379	TNF	-0.53232207
380	TLR5	-0.99925450
381	FAS	0.09013685
382	FASL	0.92726904
383	P53	-1.48113647
384	IKBKB	0.505167076
385	Case	CR25
386	TLR4	1.21431105
387	TWIST1	2.62719229
388	TLR2	2.17336304
389	TLR7	0.09079613
390	NFKB1	-0.21090343
391	ELANE	2.825430063
392	MPO	0.41060157
393	GCR	3.33952631
394	KRAS	0.98556896

395	TNF	0.61683418
396	TLR5	-0.61492445
397	FAS	-1.99764842
398	FASL	-0.15718572
399	P53	0.07285287
400	IKBKB	-2.061456747
401	Case	CR26
402	TLR4	0.14698725
403	TWIST1	-0.55301936
404	TLR2	-0.81125540
405	TLR7	2.49925679
406	NFKB1	3.34156790
407	ELANE	0.751254260
408	MPO	0.20090513
409	GCR	0.03416019
410	KRAS	2.03486147
411	TNF	-0.04502768
412	TLR5	-0.17351294
413	FAS	0.18381280
414	FASL	-1.35408317
415	P53	-1.91352347
416	IKBKB	-1.273856069
417	Case	CR27
418	TLR4	2.36372914
419	TWIST1	2.75113189
420	TLR2	2.75087641
421	TLR7	3.48379017
422	NFKB1	-0.24774763
423	ELANE	1.820368859
424	MPO	-0.36723892
425	GCR	0.91024786
426	KRAS	-0.15652828
427	TNF	2.73206257
428	TLR5	0.65451321
429	FAS	-2.15891621
430	FASL	-0.75755730
431	P53	-2.03065845
432	IKBKB	-0.815185238
433	Case	CR28
434	TLR4	-0.69866310
435	TWIST1	1.28120713
436	TLR2	2.35093401
437	TLR7	-0.86847257
438	NFKB1	0.75600575

439	ELANE	3.468022713
440	MPO	-0.65587403
441	GCR	1.79955697
442	KRAS	1.82731242
443	TNF	0.25184842
444	TLR5	0.63394880
445	FAS	-0.86264272
446	FASL	0.05100667
447	P53	0.77018233
448	IKBKB	-1.716161011
449	Case	CR29
450	TLR4	1.32615615
451	TWIST1	-0.57993896
452	TLR2	0.21816052
453	TLR7	-0.20210118
454	NFKB1	1.93586087
455	ELANE	1.704619285
456	MPO	0.07516186
457	GCR	3.26560644
458	KRAS	2.46530833
459	TNF	-0.37302067
460	TLR5	-0.71961459
461	FAS	-1.43875510
462	FASL	-1.29074468
463	P53	-0.26902073
464	IKBKB	-1.377418032
465	Case	CR30
466	TLR4	2.01936452
467	TWIST1	-0.52343897
468	TLR2	3.02104546
469	TLR7	2.21026226
470	NFKB1	-0.05774033
471	ELANE	3.193286139
472	MPO	2.90757481
473	GCR	2.43210202
474	KRAS	2.47010841
475	TNF	-0.35343804
476	TLR5	1.72056378
477	FAS	0.99992923
478	FASL	0.37878184
479	P53	-0.30977395
480	IKBKB	-0.694693767
481	Case	CR31
482	TLR4	-0.20058494

483	TWIST1	2.32623775
484	TLR2	2.92420292
485	TLR7	2.29834789
486	NFKB1	1.28386083
487	ELANE	2.588147438
488	MPO	3.03632227
489	GCR	-0.15308277
490	KRAS	-0.11909661
491	TNF	1.68656603
492	TLR5	-0.67223278
493	FAS	0.66315863
494	FASL	0.84639040
495	P53	0.46782031
496	IKBKB	-0.380537026
497	Case	CR32
498	TLR4	2.07161684
499	TWIST1	0.49027457
500	TLR2	2.08540385
501	TLR7	-0.07046730
502	NFKB1	2.94993429
503	ELANE	2.313198432
504	MPO	1.83010650
505	GCR	3.32159981
506	KRAS	0.14123436
507	TNF	-0.56153306
508	TLR5	-0.10509159
509	FAS	-0.16160331
510	FASL	0.24487897
511	P53	0.90781418
512	IKBKB	-0.338627279
513	Case	CR33
514	TLR4	1.88541973
515	TWIST1	-0.52814760
516	TLR2	0.48060327
517	TLR7	3.31257107
518	NFKB1	1.20630885
519	ELANE	-0.835479638
520	MPO	2.62650044
521	GCR	-0.01936750
522	KRAS	-0.30032743
523	TNF	0.96100580
524	TLR5	0.15230126
525	FAS	0.84738177
526	FASL	-1.26953621

527	P53	-0.62598614
528	IKBKB	-1.808931093
529	Case	CR34
530	TLR4	1.35451742
531	TWIST1	2.41818137
532	TLR2	1.77238071
533	TLR7	1.06822339
534	NFKB1	-0.29008608
535	ELANE	1.894641702
536	MPO	3.37915944
537	GCR	0.92857771
538	KRAS	1.95369571
539	TNF	0.76152267
540	TLR5	-0.44486944
541	FAS	-0.23780702
542	FASL	-0.78588475
543	P53	0.54165449
544	IKBKB	0.590722483
545	Case	CS1
546	TLR4	-1.91617819
547	TWIST1	0.21703582
548	TLR2	0.63208493
549	TLR7	-1.05759056
550	NFKB1	-1.60600234
551	ELANE	-1.670798733
552	MPO	0.44582402
553	GCR	-1.19730863
554	KRAS	-0.11408166
555	TNF	-1.53079166
556	TLR5	0.11081331
557	FAS	0.01610904
558	FASL	0.04612262
559	P53	0.52558018
560	IKBKB	0.781607668
561	Case	CS2
562	TLR4	0.79064137
563	TWIST1	-0.98791178
564	TLR2	-0.36810504
565	TLR7	-0.87750903
566	NFKB1	-0.07458511
567	ELANE	-1.829979494
568	MPO	-1.43190058
569	GCR	-0.85537168
570	KRAS	0.18653482

571	TNF	-0.69888010
572	TLR5	-0.65677541
573	FAS	0.06864689
574	FASL	0.03285200
575	P53	0.52769821
576	IKBKB	-0.947274562
577	Case	CS3
578	TLR4	-1.17980306
579	TWIST1	-0.47191294
580	TLR2	0.35140712
581	TLR7	-1.39007637
582	NFKB1	0.77414820
583	ELANE	-0.420394886
584	MPO	-1.96732515
585	GCR	-1.46747333
586	KRAS	-0.55083493
587	TNF	0.23908286
588	TLR5	-0.03525878
589	FAS	-0.62597222
590	FASL	-0.75150608
591	P53	0.05114987
592	IKBKB	-0.364605348
593	Case	CS4
594	TLR4	-1.20747959
595	TWIST1	-1.87229378
596	TLR2	-1.11502018
597	TLR7	0.13542458
598	NFKB1	-1.59942666
599	ELANE	-1.621675825
600	MPO	-1.43304906
601	GCR	-1.87418095
602	KRAS	-1.75279755
603	TNF	0.80140364
604	TLR5	-0.43072939
605	FAS	0.22156247
606	FASL	0.63431364
607	P53	1.02573213
608	IKBKB	-0.106936762
609	Case	CS5
610	TLR4	-0.80678737
611	TWIST1	-0.91524222
612	TLR2	0.83432388
613	TLR7	-1.61513445
614	NFKB1	-0.98566762

615	ELANE	-0.027041740
616	MPO	-0.33275154
617	GCR	0.66710253
618	KRAS	-1.54539394
619	TNF	0.55516440
620	TLR5	-1.50890306
621	FAS	-0.63270525
622	FASL	-0.36613984
623	P53	0.27965714
624	IKBKB	-0.081503478
625	Case	CS6
626	TLR4	-0.52046012
627	TWIST1	0.89705746
628	TLR2	-1.72940907
629	TLR7	-1.48704529
630	NFKB1	-1.31309310
631	ELANE	0.712510907
632	MPO	-1.49995383
633	GCR	-1.13420338
634	KRAS	-1.09756596
635	TNF	-1.87275564
636	TLR5	-0.31439299
637	FAS	0.41996953
638	FASL	0.53631804
639	P53	-0.21611255
640	IKBKB	-0.607721753
641	Case	CS7
642	TLR4	-0.13522719
643	TWIST1	0.56979981
644	TLR2	-0.28240124
645	TLR7	0.32297808
646	NFKB1	-1.72666511
647	ELANE	-1.095487594
648	MPO	-1.40809356
649	GCR	-1.05866018
650	KRAS	0.67648840
651	TNF	-1.65415954
652	TLR5	-1.06590585
653	FAS	-0.80290371
654	FASL	1.02994108
655	P53	0.93078688
656	IKBKB	1.233548817
657	Case	CS8
658	TLR4	-0.46086511

659	TWIST1	0.11275207
660	TLR2	0.07167195
661	TLR7	-0.47729720
662	NFKB1	-0.49942986
663	ELANE	-1.894723778
664	MPO	0.16832369
665	GCR	-0.65574806
666	KRAS	-0.14376994
667	TNF	0.41165935
668	TLR5	0.93723883
669	FAS	0.59149884
670	FASL	-0.12560480
671	P53	-0.71474772
672	IKBKB	1.189507729
673	Case	CS9
674	TLR4	0.05137840
675	TWIST1	0.63661488
676	TLR2	-1.23140043
677	TLR7	-1.55721354
678	NFKB1	0.64903691
679	ELANE	-0.796901744
680	MPO	-0.40897787
681	GCR	-0.81076882
682	KRAS	0.58927054
683	TNF	0.18523866
684	TLR5	-0.50168332
685	FAS	-0.83418128
686	FASL	1.00426551
687	P53	-0.69722286
688	IKBKB	1.096094704
689	Case	CS10
690	TLR4	0.63720970
691	TWIST1	0.20227326
692	TLR2	-1.54234076
693	TLR7	-1.40871170
694	NFKB1	0.78359527
695	ELANE	0.005388105
696	MPO	-0.67916926
697	GCR	0.24758493
698	KRAS	0.79749209
699	TNF	-0.57404087
700	TLR5	0.28898534
701	FAS	1.06830448
702	FASL	1.03344012

703	P53	0.10573464
704	IKBKB	0.406900958
705	Case	CS11
706	TLR4	0.91570299
707	TWIST1	0.34058798
708	TLR2	0.79231152
709	TLR7	0.45723103
710	NFKB1	-0.21987084
711	ELANE	0.959635089
712	MPO	0.32735930
713	GCR	0.57685039
714	KRAS	-1.20439277
715	TNF	-0.60266507
716	TLR5	0.24930949
717	FAS	-0.51940477
718	FASL	0.15869162
719	P53	-0.94189508
720	IKBKB	-0.570522750
721	Case	CS12
722	TLR4	-0.98285515
723	TWIST1	-0.50508538
724	TLR2	-1.26040164
725	TLR7	-0.53851589
726	NFKB1	0.09973467
727	ELANE	-1.623950480
728	MPO	-0.87461525
729	GCR	-0.47836682
730	KRAS	0.06831245
731	TNF	0.37901915
732	TLR5	-0.41087490
733	FAS	0.79090381
734	FASL	-0.44944342
735	P53	-0.04419648
736	IKBKB	-0.900111783
737	Case	NC1
738	TLR4	-0.87891891
739	TWIST1	-1.30618983
740	TLR2	0.37152646
741	TLR7	-1.17612846
742	NFKB1	0.23624269
743	ELANE	-0.189718273
744	MPO	-1.39407892
745	GCR	0.82905126
746	KRAS	-1.90783721

747	TNF	-1.02184732
748	TLR5	0.72832850
749	FAS	0.87156890
750	FASL	0.70256997
751	P53	1.15186593
752	IKBKB	0.213092722
753	Case	NC2
754	TLR4	-0.62565554
755	TWIST1	-1.90765719
756	TLR2	-1.71965026
757	TLR7	-0.45272755
758	NFKB1	-1.72189575
759	ELANE	0.551224969
760	MPO	-0.69881195
761	GCR	-1.60188065
762	KRAS	-1.51884118
763	TNF	0.03957763
764	TLR5	-0.37183645
765	FAS	0.26909745
766	FASL	-0.52980807
767	P53	1.25441649
768	IKBKB	-0.006637377
769	Case	NC3
770	TLR4	-1.15376825
771	TWIST1	-1.57354893
772	TLR2	-0.91818135
773	TLR7	-1.85011531
774	NFKB1	0.19740610
775	ELANE	-1.701890782
776	MPO	-0.47747884
777	GCR	-0.66000399
778	KRAS	-1.95502358
779	TNF	0.38079496
780	TLR5	-0.68666972
781	FAS	0.25839576
782	FASL	-0.85135032
783	P53	-0.35521648
784	IKBKB	1.275251247
785	Case	NC4
786	TLR4	-1.52555965
787	TWIST1	-1.12857708
788	TLR2	0.66251322
789	TLR7	-1.30263693
790	NFKB1	0.32108073

791	ELANE	0.899099471
792	MPO	0.88821532
793	GCR	0.77920350
794	KRAS	0.61235804
795	TNF	-0.20768564
796	TLR5	-1.06608456
797	FAS	-0.14359508
798	FASL	0.01540689
799	P53	-0.03875096
800	IKBKB	1.474491294
801	Case	NC5
802	TLR4	0.56754905
803	TWIST1	-1.54958554
804	TLR2	-0.43901643
805	TLR7	-0.97015498
806	NFKB1	-1.55395991
807	ELANE	0.252097492
808	MPO	-0.60330739
809	GCR	-0.40120933
810	KRAS	0.09742885
811	TNF	-0.20044751
812	TLR5	-1.56852562
813	FAS	-0.73469211
814	FASL	0.58792331
815	P53	1.38061895
816	IKBKB	1.255136750
817	Case	NC6
818	TLR4	-1.88813912
819	TWIST1	0.48116058
820	TLR2	-0.36080209
821	TLR7	0.19836664
822	NFKB1	-1.48759102
823	ELANE	0.456315948
824	MPO	-1.59688306
825	GCR	-0.09053130
826	KRAS	0.21881552
827	TNF	-1.90593025
828	TLR5	-0.28653885
829	FAS	-0.65574564
830	FASL	-0.25996285
831	P53	0.97874239
832	IKBKB	1.372863318
833	Case	NC7
834	TLR4	-0.35259296

835	TWIST1	-1.78195351
836	TLR2	0.80510938
837	TLR7	-1.85757360
838	NFKB1	0.24157416
839	ELANE	-1.453589589
840	MPO	-1.21589968
841	GCR	0.51937020
842	KRAS	-1.38443083
843	TNF	-0.64409009
844	TLR5	-0.15260244
845	FAS	1.36284886
846	FASL	-0.32064879
847	P53	1.01125845
848	IKBKB	-0.811012844
849	Case	NC8
850	TLR4	0.67058970
851	TWIST1	0.04054484
852	TLR2	-0.71318395
853	TLR7	0.04815973
854	NFKB1	-1.51101505
855	ELANE	-1.572730650
856	MPO	-0.35744430
857	GCR	-1.57974926
858	KRAS	-1.69696374
859	TNF	0.37842296
860	TLR5	-0.28756756
861	FAS	1.45513517
862	FASL	1.17727865
863	P53	-0.75886827
864	IKBKB	0.279848280
